# Efficacy and safety of intravenous daratumumab-based treatments for AL amyloidosis: a systematic review and meta-analysis

**DOI:** 10.1186/s12935-022-02635-6

**Published:** 2022-07-04

**Authors:** Chunyan Sun, Xiaohong Wang, Renyi Zhang, Lingjie Xu, Bin Wang, Jian Li

**Affiliations:** 1 Institute of Hematology, Union Hospital, Tongji Medical College, Huazhong University of Science and Technology, Wuhan, Hubei China; 2 Xi’an Janssen Pharmaceutical Ltd., Shanghai, China; 3 Department of Hematology, Peking Union Medical College Hospital, Chinese Academy of Medical Sciences and Peking Union Medical College, No. 1 Shuaifuyuan, Wangfujing, Dongcheng, Beijing, 100730 China; 4 Xi’an Janssen Pharmaceutical Ltd., Beijing, China

**Keywords:** Intravenous daratumumab, Immunoglobulin light-chain amyloidosis, Efficacy, Safety, Meta-analysis

## Abstract

**Background:**

Intravenous daratumumab (DARA IV) has been increasingly used in the treatment of amyloid light-chain (AL) amyloidosis. However, the outcomes for patients administered with DARA IV have not been aggregated. The objective of this systematic review and meta-analysis was to investigate the efficacy and safety of DARA IV for AL amyloidosis.

**Methods:**

We searched Medline, EMBASE, Cochrane Library and Web of Science up to 17 June 2021. Response rates and survival rates, and the corresponding 95% confidence intervals (CIs) were pooled and calculated using a fixed-effects model.

**Results:**

Thirty studies (5 cohort studies and 25 single-arm studies) with 997 patients were included. In patients receiving DARA IV-based treatments, very good partial response or better response rate, complete response rate, very good partial response rate, partial response rate and overall response rate were 66% (95% CI, 62–69%), 30% (95% CI, 23–36%), 40% (95% CI, 33–46%), 17% (95% CI, 14–21%), and 77% (95% CI, 73–80%), respectively. Cardiac and renal responses were 41% (95% CI, 34–49%) and 43% (95% CI, 32–54%), respectively. 58% (95% CI, 49–66%) of patients achieved PFS one year or longer. 2.5% (range, 1–10.0%) of patients experienced grade 3 or 4 adverse events, of which the most common adverse event was lymphocytopenia (range, 13.6–25.0%).

**Conclusion:**

This study supports the efficacy and safety of DARA IV for the treatment of patients with AL amyloidosis.

**Supplementary Information:**

The online version contains supplementary material available at 10.1186/s12935-022-02635-6.

## Introduction

Immunoglobulin light chain (AL) amyloidosis is characterized by a clonal population of bone marrow plasma cells that produces a κ or λ type monoclonal light protein chain as either an intact molecule or a fragment. This insoluble protein deposits in tissues and interferes with organ function [1]. Treatment of systemic AL amyloidosis relies primarily on multiple myeloma regimens by suppressing the secretion of amyloid-forming monoclonal free light chains (FLCs) by underlying plasma cell clone [2]. Autologous stem cell transplantation (ASCT) is the preferred regimen for patients with transplant-eligible AL Amyloidosis. Previous studies revealed that the median overall survival (OS) of patients with AL amyloidosis who received ASCT was 10 years, although most patients were not eligible for this therapy [3, 4]. For patients who are ineligible for ASCT, therapeutic regimens derived from multiple myeloma treatments are usually used for AL Amyloidosis, such as bortezomib-cyclophosphamide-dexamethasone (CyBorD) [5, 6]. Moreover, it has been shown that the hematological complete remission (CR) rates of newly diagnosed patients receiving CyBorD regimen were 23–47% and the prognosis of stage IIIB patients were still poor [7]. CR rates of lenalidomide-based therapies for newly diagnosed patients were only 14–23% [8, 9]. Although the survival could be improved by the aforementioned therapeutic regimens in some patients with AL Amyloidosis [10], some patients are still unable to benefit from these treatments, particularly those with an advanced cardiac disease [11]. Therefore, new therapies are in need for the treatments of AL amyloidosis patients.

Daratumumab is the first-in-class antibody-based therapy targeting the glycoprotein CD38, which is overly expressed on the surface of abnormal plasma cells [12, 13]. It has been reported that a higher CD38 expression is associated with adverse survival in AL amyloidosis [14]. Plasma cell death can be induced by daratumumab through various mechanisms, including complement-dependent cytotoxicity, antibody-dependent cell-mediated cytotoxicity, antibody-dependent cellular phagocytosis, and direct cellular apoptosis [13]. The combination of subcutaneous daratumumab with bortezomib, cyclophosphamide, and dexamethasone has been demonstrated to be effective and safe for patients with AL amyloidosis in the Andromeda study [15]. However, previously published original studies on intravenous daratumumab (DARA IV)-based regimens are hampered by the difference of study populations and relatively small sample sizes [16, 17] and there has been a lack of systematic assessment and synthesis of the efficacy and safety of DARA IV-based in AL amyloidosis. Therefore, this study used meta-analysis to synthesize the efficacy and safety of DARA IV-based therapies in the treatment of patients with AL Amyloidosis to provide reference for the selection of treatment in clinical settings.

## Materials and methods

This systematic review and meta-analysis was performed in accordance with the Preferred Reporting Items for Systematic Review and Meta-Analysis (PRISMA 2020) extension statement [18]. The protocol for this systematic review was registered in the International Platform of Registered Systematic Review and Meta-analysis Protocols (INPLASY) with an identification number “INPLASY202160054”.

### Data sources and literature searches

Systematic literature searches were conducted in Medline, EMBASE, Cochrane Library and Web of Science with the search terms relating to DARA IV and amyloidosis. Searches were performed from the database inception to June 17, 2021 and were restricted to full papers using human subjects and published in English. All document types were included in the screening phase. A detailed search strategy is available in the Additional file 1: Methods S1. To supplement the electronic searches, reference lists of included studies was checked for relevant studies to identify additional published or unpublished materials (grey literature).

### Study selection

Two reviewers independently screened studies by viewing the titles and abstracts. All potentially relevant citations were requested and inspected in detail using the full-text version. Disagreements were resolved by discussion, with assistance from a third party if necessary. A PRISMA flow diagram was constructed to show the full study selection process. The following inclusion and exclusion criteria were used to select studies at title and abstracts stage as well as full-text screening stage: eligible study design includes interventional and noninterventional studies investigating the efficacy and safety of DARA IV-based therapy for the treatment of patients with AL amyloidosis. For patients with AL amyloidosis, there was no limitation on age, gender, ethnicity, prior lines of therapy, Mayo stage, or comorbidity. The administration of daratumumab was limited to intravenous use. The following studies were excluded: studies not reported in English, studies without outcome data, and case reports.

### Outcomes

The primary outcome was a very good partial response or better response (≥ VGPR) rate, defined as CR or VGPR. The secondary outcomes including CR was defined as normalization of the difference between involved and uninvolved free light chain (dFLC) levels and ratio, negative serum and urine immunofixation, or as defined in the original studies; VGPR was defined as a reduction in dFLC to < 40 mg/L, or as defined in the original studies; Partial response rate (PR) was defined as a greater than 50% reduction in the dFLC, or as defined in the original studies; Overall response rate (ORR) was defined as the sum rate of patients with CR, VGPR, and PR; Time to hematologic response or best hematologic response; Cardiac response rate was defined as an N-terminal pro-brain natriuretic peptide (NT-proBNP) response (> 30% and > 300 ng/L decrease in patients with a baseline NT-proBNP > 650 ng/L) or New York Heart Association (NYHA) class response (> 2 class decrease in subjects with baseline NYHA class 3 or 4), or as defined in the original studies; Renal response rate was defined as ≥ 30% decrease in proteinuria or drop in proteinuria below 0.5 g/24 h in the absence of renal progression, or as defined in the original studies; Progression-free survival (PFS) and OS, both were defined as in the original studies of 1 year or more than 1 year; Rates of ≥ 5% grade 3 or 4 adverse events (AEs); Rates of infusion-related reactions (IRRs).

When studies reported the same hematologic response, organ response or survival of different timepoints, we chose the clinical endpoints with longest observational time.

### Data extraction

Data from each study was extracted independently by two reviewers using a standardized data extraction form, and disagreements were resolved by discussion or by referral to a third reviewer if necessary. If multiple publications were reported based on the same study population, we extracted all data from companion studies and removed duplicated data. A PICOS (Population, Intervention, Comparison, Outcome, Study design) structure was used to formulate the data extraction as follows: (1) General study characteristics: the first author’s name, the published year, country, study center (single/multiple); (2) Characteristics of participants: the number, gender and age of patients, diagnostic criteria, diagnostic results, inclusion/exclusion criteria, prior lines of therapies, Mayo stage, organ involvement, dFLC, and epidermal growth factor receptor (eGFR); (3) Interventions: treatment frequency, dosage, and treatment duration; (4) Outcomes: types of outcomes, definitions, and measurement timepoints; (5) Results: all relevant dichotomous results; (6) Study designs: randomized control trials (RCTs), non-randomized studies of interventions (NRSIs), single-arm studies, and case series.

### Quality assessment

This single-arm meta-analysis was to pool data derived from single-arm study or the Dara-based treatment arm of randomized controlled trials and cohort studies. Therefore, we chose “Quality Assessment Tool for Before-After (Pre-Post) Studies With No Control Group” that developed by the National Heart, Lung, and Blood Institute (NHLBI) and adapted for the purpose of our study (Additional file 1: Table S1) [19]. Two independent reviewers evaluated the quality of studies. The two reviewers resolved disagreements by discussion and if required, a third reviewer arbitrated.

### Data synthesis and analysis

For data expressed as median and interquartile range (IQR)/range, we narratively described the data. Fixed-effects meta-analysis was performed to synthesize data using the R package “meta” (R software version 4.0.2) [20]. For dichotomous variables, we calculated risk ratios (RRs) with 95% confidence intervals (CIs). When no event was observed, we added a fixed value (typically 0.5) to the event number of intervention group. Where heterogeneity was significant (P ≤ 0.1 and I^2^ ≥ 50%), and the sources of heterogeneity were identified, we conducted a subgroup analysis to pool the data. When the source of heterogeneity was not identified by subgroup analysis, we used a random-effects model to pool the result. We performed a subgroup analysis on the primary outcome according to the type of regimen (daratumumab ± dexamethasone versus triple regimens), line of therapy (newly diagnosed versus relapsed/refractory), Mayo stage (I versus II versus III), and primary versus secondary patients. We did a sensitivity analysis, the Freeman-Tukey double arcsine transformation analysis was performed to deal 0 event, using stata package “metaprop” (Stata software version 15.0) [21]. We also did a sensitivity analysis, random-effects meta-analysis was performed when heterogeneity was not significant (P > 0.1 and I^2^ < 50%).

## Results

### Results of study selection

**Fig. 1:**
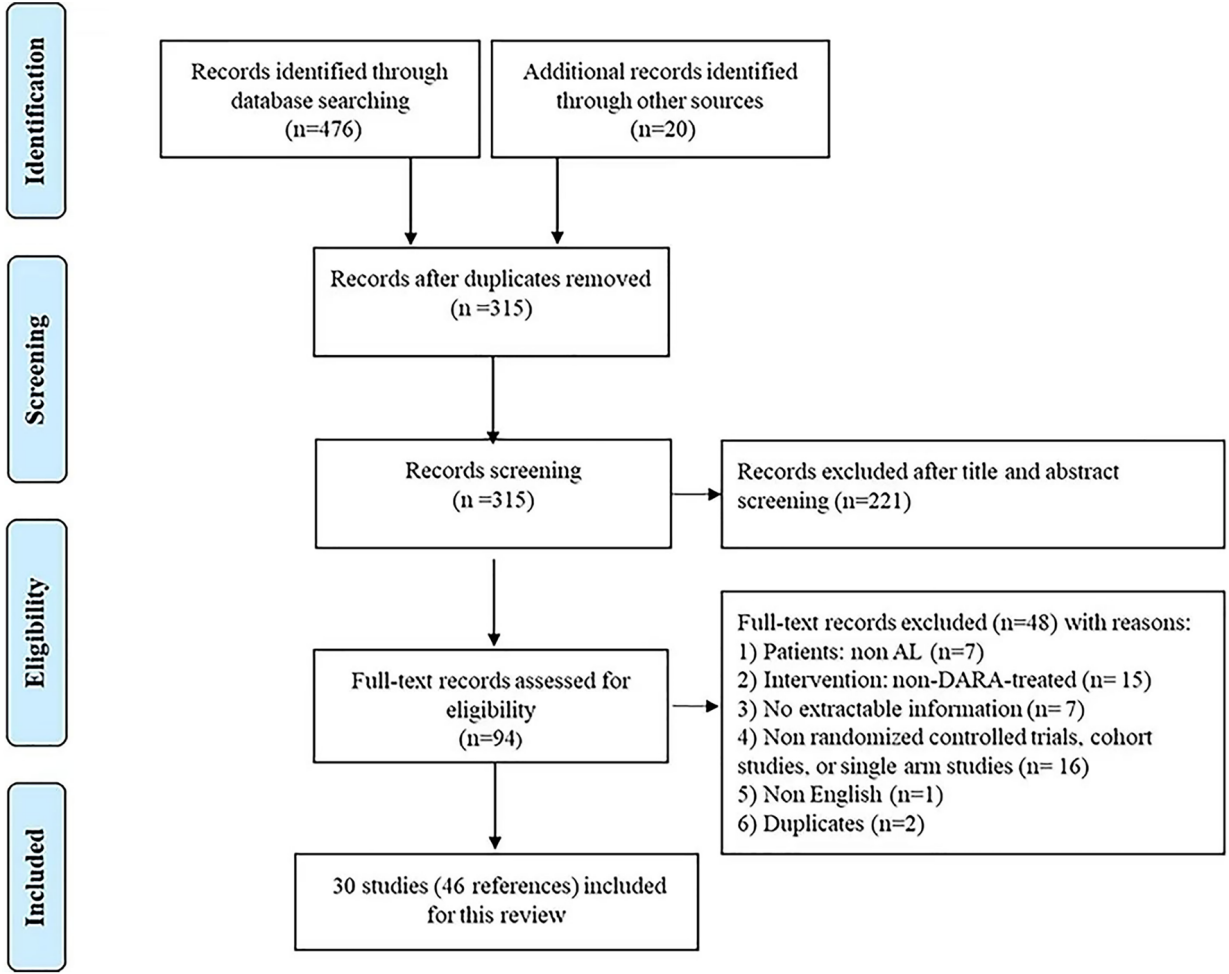
Flow diagram for identification of relevant studies

Figure 1 shows the flow diagram displaying the literature search results. The initial search retrieved a total of 496 articles, including 476 articles from databases, and 20 articles from conferences. After deduplication, 315 unique articles remained. We excluded 221 articles by screening the titles and abstracts. 48 articles were further excluded in a full-text review, leaving 46 eligible publications from 30 study populations, including 5 cohort studies and 25 single-arm studies [2, 16, 17, 22–64].

### Characteristics of included studies and participants

A total of 30 studies with 997 participants were included in this review (for details of included studies, see Additional file 1: Table S2). The data of these studies were contributed by ten countries, namely the US (n = 15), Italy (n = 3), Germany (n = 2), Israel (n = 2), France (n = 2), Switzerland (n = 1), Austria (n = 1), Spain (n = 1), the UK (n = 1) and Greece (n = 1). There was one international collaborative study (France and Italy).

The characteristics of patients included in this study are summarized in Table 1. Of 888 patients, over half (62.7%) were male; of 736 patients, the ages ranged from 34 to 91 years. Of 441 patients, 87.8% patients were λ isotype and 12.2% were κ isotype. Of patients that reported the status of disease, most of them were at the relapsed or refractory stage (489/517). Mayo 2004 cardiac staging was available for 303 patients: 16.2% were stage I, 40.6% were stage II, and 43.2% were stage III. Mayo 2012 cardiac staging was available for 109 patients: 12.8% were stage I, 33.0% were stage II, 30.3% were stage III, and 23.9% were stage IV. The most commonly involved organs included the heart (74.3%), kidney (60.5%) and liver (8.2%).

**Table 1:** Summary information of the characteristics of participants from included studies contributing to statistical analyses

Characteristics	No. of patients, or range of median
Gender, n (%) (n_total_ = 888)	
Male	557 (62.7)
Female	331 (37.3)
Age (years) (n_total_ = 736)	
Range	34–91
Newly diagnosed or relapsed/refractory, n (%) (n_total_ = 997)	
Newly diagnosed	28 (2.8)
Relapsed/refractory	489 (49.0)
Mixed population	254 (25.5)
Not reported	226 (22.7)
AL isotype, n (%) (n_total_ = 441)	
λ	387 (87.8)
k	54 (12.2)
Mayo 2004 Stage, n (%) (n_total_ = 303)	
I	49 (16.2)
II	123 (40.6)
III	50 (16.5)
IIIA	60 (19.8)
IIIB	21 (6.9)
Mayo 2012 Stage, n (%) (n_total_ = 109)	
I	14 (12.8)
II	36 (33.0)
III	33 (30.3)
IV	26 (23.9)
Involved organs, n (%) (n_total_ = 936)	
Heart	688 (74.3)
Kidney	560 (60.5)
Liver	76 (8.2)
Others*	34 (3.7)
eGFR (mL/min/m^2^) (n_total_ = 330)	
Range of median	34–73
dFLC (mg/L) (n_total_ = 711)	
Range of median	34.5–276.9

*dFLC*  difference between the involved and uninvolved light chain, *eGFR*  epidermal growth factor receptor

^*^Pulmonary, tongue, bone marrow, muscle, spleen, upper aerodigestive tract

### Treatments

Of 997 patients with identified therapies, 665 (n = 665/977, 66.7%) received a daratumumab mono or combined with dexamethasone regimen (Dara ± dex), Daratumumab + Bortezomib + Dexamethasone (DVd) in 108 (10.8%), Daratumumab + Lenalidomide + Dexamethasone (DRd) in 71 (7.1%) and Daratumumab + Cyclophosphamide + Dexamethasone (DCd) in 4 (0.4%) (Table 2). ﻿The median of the treatment duration ranged from 4 to 31 cycles.﻿

**Table 2:** Distribution of treatment regimens

Treatment regimens	n of patients	Rate (%)
Dara ± dex	665	66.7
DVd	108	10.8
DRd	71	7.1
DCd	4	0.4
Mixed Daratumumab-based treatments^*^	149	15.0

*Dara ± dex*  Daratumumab ± Dexamethasone, *DVd*  Daratumumab + Bortezomib + Dexamethasone, *DRd*  Daratumumab + Lenalidomide + Dexamethasone, *DCd*  Daratumumab + Cyclophosphamide + Dexamethasone

^*^Mixed Daratumumab-based treatments indicate there were more than one kind of daratumumab-based treatment in the study (daratumumab was combined with dexamethasone, bortezomib, lenalidomide, pomalidomide, cyclophosphamide, ixazomid or carfilzomib)

### Quality assessment of included studies

All 30 studies clearly stated research questions or objectives, of which 27 studies clearly prespecified and described eligibility/selection criteria for the study population. The participants lost to follow-up were less than 20% in 26 studies, while in the other four studies more than 20% of participants were lost to follow-up. Eighteen studies prespecified and clearly defined measures of outcomes. Only one study described the evaluable sample size, while in the other studies, the authors were uncertain whether sample sizes were sufficient. None of the 30 studies reported blinding of the participants. Details of the quality assessment of included studies are shown in Additional file 1: Table S1.

### Hematologic response and organ response

**Fig. 2:**
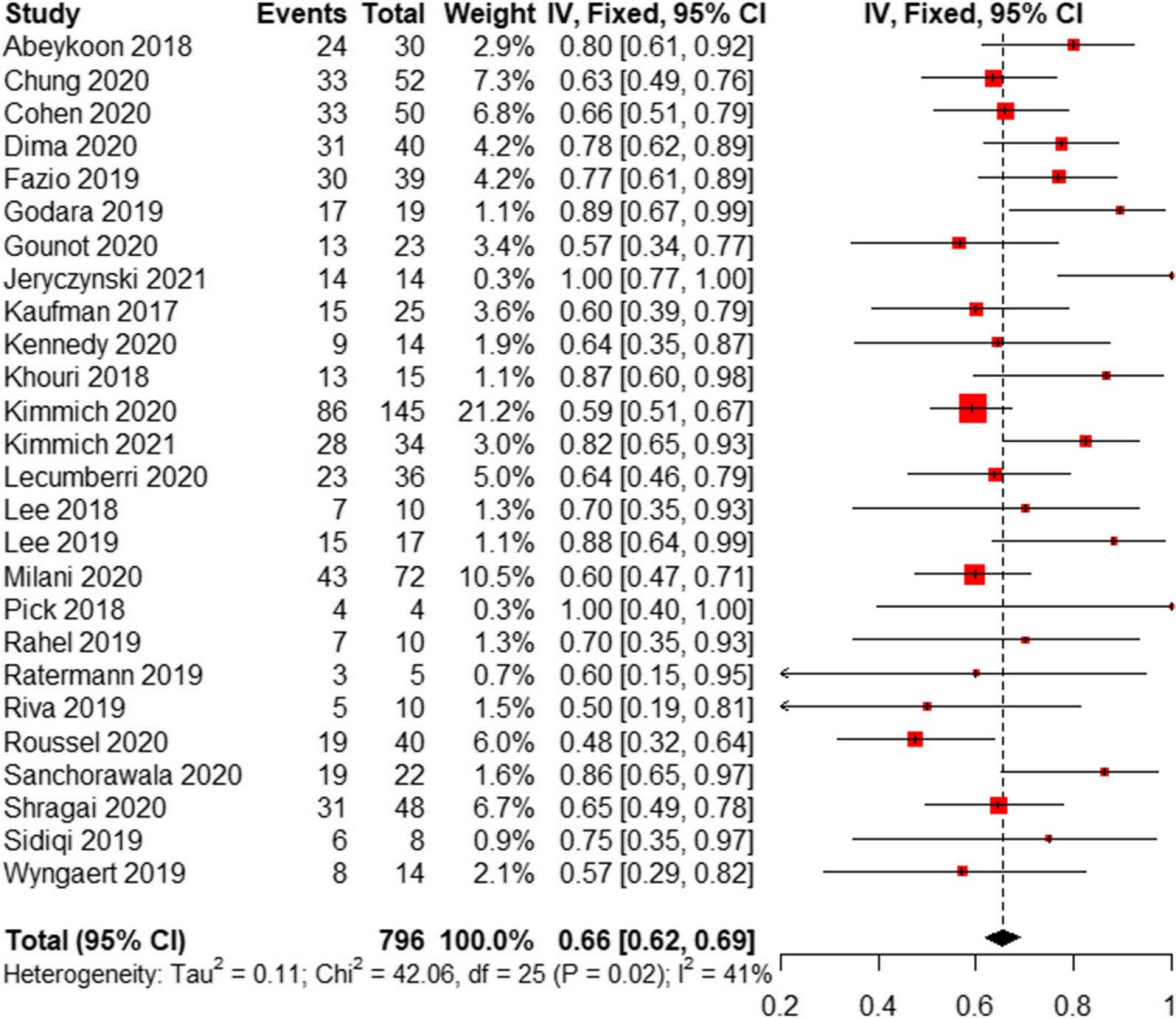
Meta-analysis forest plot of very good partial response or better

 In 26 studies that reported a robust hematologic response (Fig. 2), 536 of 769 patients (66%; 95% CI, 62–69%; I^2^ = 41%) achieved a ≥ VGPR after treatment with daratumumab-based regimens. The overall response rate was 77% (95% CI, 73–80%), with a CR was achieved in 30% of patients (95% CI, 23–36%), a VGPR was achieved in 40% (95% CI, 33–46%), a PR was achieved in 17% (95% CI, 14–21%). In 15 studies with 397 patients, the median time to a first hematologic response ranged from 7 to 78 days, and in 11 studies with 253 patients, the median time to the best hematologic response ranged from 30 to 336 days (Additional file 1: Tables S3, S4). Cardiac and renal responses occurred in 41% (95% CI, 34–49%) and 43% (95% CI, 32–54%) of patients, respectively (Table 3). Forest plots are presented in Additional file 1: Fig. S1-S6.

**Table 3:** Hematological and organ responses to daratumumab-based regimens

Secondary outcome	Studies (n)	Responses (n)	Patients (n)	Response rate % (95% CI)		I^2^
*Hematologic response*						
CR	24	193	752	30 (23, 36)		65%
VGPR	23	295	729	40 (33, 46)		61%
PR	18	82	544	17 (14, 21)		50%
ORR	27	633	806	77 (73, 80)		45%
*Organ response*						
Cardiac response	20	166	440	41 (34, 49)		56%
Renal response	18	139	345	43 (32, 54)		69%

*CI*  confidence interval, *CR*  complete response, *PR*  partial response rate, *VGPR*  very good partial response

### Very good partial response or better rate

**Fig. 3:**
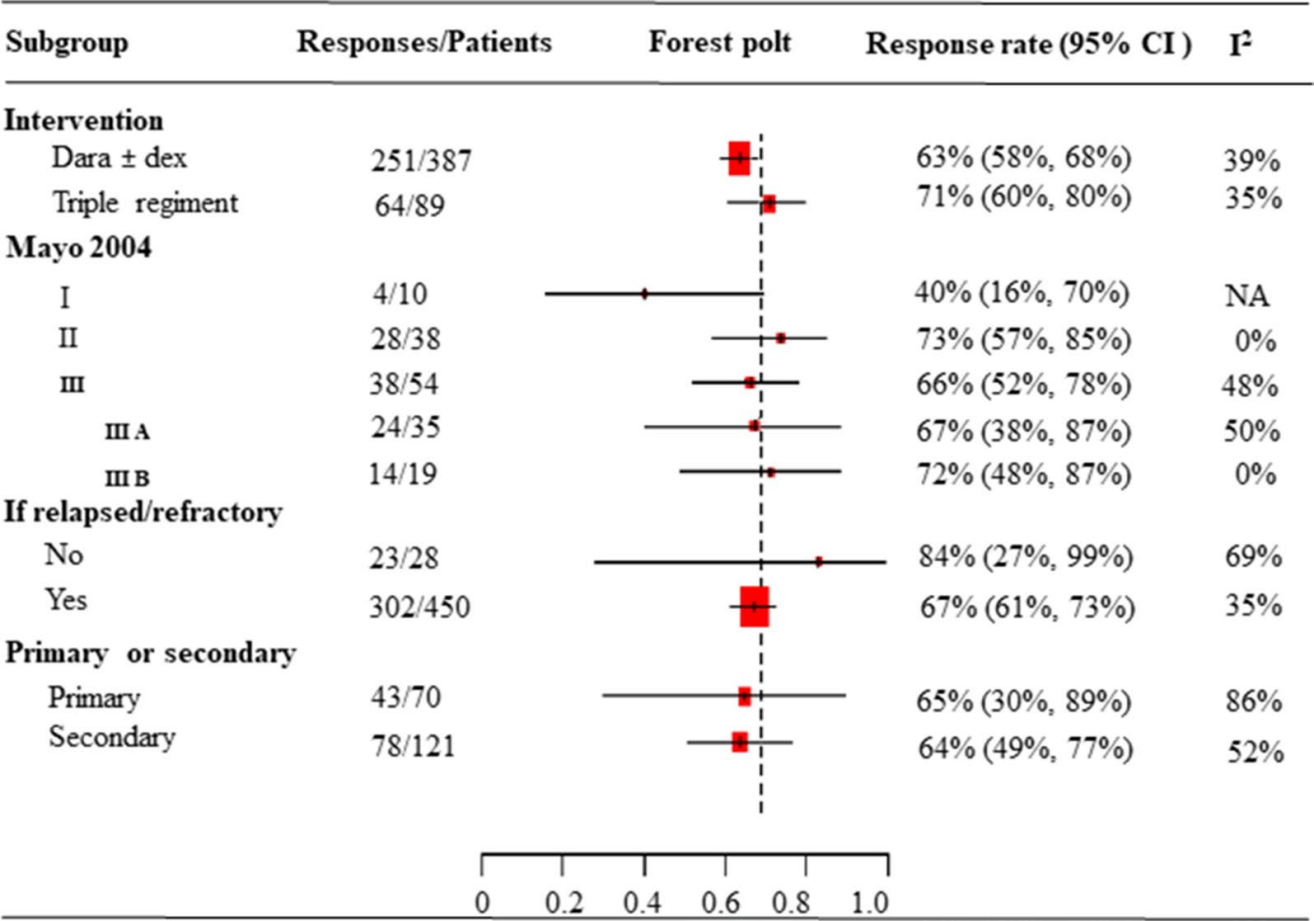
Forest plot of pooled ≥ VGPR rate by subgroups

Figure 3 shows the pooled ≥ VGPR rate by categories of treatments, Mayo 2004 stage, newly diagnosis or relapsed/refractory and primary or secondary AL amyloidosis. Subgroup analyses revealed improvement in the ≥ VGPR rate following treatment with triple regimens. In three studies, 64 of 89 patients treated with a daratumumab-based triple regimens achieved ≥ VGPR (71%; 95% CI, 60–80%; I^2^ = 35%), whereas 251 of 387 patients in 16 studies treated with daratumumab mono or combined with dexamethasone (Dara ± dex) achieved ≥ VGPR (63%; 95% CI, 58–68%; I^2^ = 39%) (Fig. 3, Additional file 1: Fig. S7). It also showed that similar ≥ VGPR rates across different Mayo stages (Fig. 3, Additional file 1: Fig. S8-S9,) and patients with primary or secondary AL amyloidosis (Fig. 3, Additional file 1: Fig. S10), whereas a higher ≥ VGPR rates were observed in newly diagnosed patients than in patients with relapsed/refractory disease (84% vs. 67%, respectively) (Fig. 3, Additional file 1: Fig. S11). Notably, interpretation of subgroup analysis result should made with caution because of the small and imbalance sample sizes in each group.

Meta-analysis with a random effects model showed heterogeneity in the rate of VGPR, CR and renal response and the source of heterogeneity could not be identified when investigated by predefined subgroup factors. Line of therapy of newly diagnosed or relapsed/refractory may be one of the sources of heterogeneity in cardiac response rate. Subgroup analysis of the cardiac response in the newly diagnosed group showed ten of the 18 (55%; 95% CI, 32–76%; I^2^ = 12%) patients had a cardiac response, while in the relapsed or refractory group, 77 of 200 (39%; 95% CI, 32–46%; I^2^ = 36%) patients had a cardiac response. However, due to the small sample size of the subgroup, the results must be interpreted with caution.

### Progression-free survival and overall survival

Three studies [2, 48, 64] reported a PFS rate of 1 year or longer. Seventy-three of 126 (58%; 95% CI, 49–66%; I^2^ = 46%) patients reached PFS after 1 year or longer (Additional file 1: Fig. S12). Of 11 studies reporting OS, 411 of 534 (76%; 95% CI, 72–80%; I^2^ = 31%) patients survived after 1 year or longer (Additional file 1: Fig. S13). The causes of death were disease progression, infection, sepsis, immunomodulatory agent–related rejection of the transplanted heart and cardiac complication [2, 31, 54, 58, 64].

### Adverse events

Fifteen studies reported IRRs. Of the included 276 patients, 87 experienced grade 1 or 2 IRRs (33%; 95% CI, 21–47%; I^2^ = 76%) with a median rate of 33.3% (range, 11.1–70.0%) (Additional file 1: Fig. S14), 10 of 432 patients (3%; 95% CI, 2–6%; I^2^ = 0) experienced grade 3 or 4 IRRs with a median rate of 2.5% (range, 1.0–10.0%) (Additional file 1: Fig. S15).

Nine studies reported grade 3 or 4 adverse events. The most common (≥ 5%) grade 3 or 4 adverse events reported in more than one study were lymphocytopenia, heart failure, infection complications, pneumonia, fatigue, atrial fibrillation, neutropenia, and diarrhea. (Table 4) Other adverse events reported were presented in Additional file 1: Table S5.

**Table 4:** Most common (≥ 5%) grade 3 or 4 treatment-emergent adverse events (TEAEs) reported in more than one study

Adverse events	Study ID	Intervention	Events (n)	Total (n)	Rate (%)
Lymphocytopenia	Kimmich 2021	DRd	11	44	25.0
	Kimmich 2020	Dara ± dex, DVd	27	142	20.2
	Sanchorawala 2020	Dara ± dex	3	22	13.6
Heart failure^*^	Kimmich 2020	Dara ± dex, DVd	15	168	8.9
	Kaufman 2017	Dara ± dex	1	25	4.0
	Sanchorawala 2020	Dara ± dex	3	22	13.6
Infection complications	Cohen 2020	Dara ± dex	0	53	0.0
	Dima 2020	Mixed ^†^	3	40	7.5
	Gounot 2020	Mixed ^†^	2	25	8.0
	Kaufman 2017	Dara ± dex	2	25	8.0
	Kimmich 2020	Dara ± dex, DVd	28	168	16.0
	Milani 2020	Mixed ^†^	3	72	4.2
	Sanchorawala 2020	Dara ± dex	4	22	18.2
	Shragai 2020	Mixed ^†^	7	48	14.6
Pneumonia	Dima 2020	Mixed ^†^	2	40	5.0
	Kimmich 2021	DRd	7	44	15.9
	Milani 2020	Mixed ^†^	4	72	5.6
	Shragai 2020	Mixed ^†^	5	48	10.4
Fatigue	Cohen 2020	Dara ± dex	0	53	0.0
	Sanchorawala 2020	Dara ± dex	2	22	9.1
Atrial fibrillation	Kimmich 2020	Dara ± dex, DVd	0	168	0.0
	Milani 2020	Mixed^†^	2	72	2.8
	Sanchorawala 2020	Dara ± dex	4	22	18.2
Neutropenia	Abeykoon 2018	Dara ± dex	3	44	6.8
	Kimmich 2020	Dara ± dex, DVd	0	142	0.0
	Milani 2020	Mixed^†^	2	72	2.8
Diarrhea	Cohen 2020	Dara ± dex	0	53	0.0
	Kimmich 2020	Dara ± dex	1	168	0.6
	Sanchorawala 2020	Dara ± dex	2	22	9.1
	Shragai 2020	Mixed^†^	1	48	2.1

*Dara ± dex*  Daratumumab ± Dexamethasone, *DVd*  Daratumumab + Bortezomib + Dexamethasone

^*^Including congestive heart failure and decompensated heart failure

^†^Mixed Daratumumab-based treatments indicate there were more than one kind of daratumumab-based treatment in the study (daratumumab was combined with dexamethasone, bortezomib, lenalidomide, pomalidomide, cyclophosphamide, ixazomid, or carfilzomib)

### Sensitivity analysis

The CR was achieved in 29% of patients (95% CI, 22–36%), a VGPR was achieved in 39% (95% CI, 32–46%), a PR was achieved in 14% (95% CI, 9–19%) (Additional file 1: Fig. S16–S18). Renal responses occurred in 41% (95% CI, 30–53%) (Additional file 1: Fig. S19). 10 of 432 patients (1%; 95% CI, 0–2%; I^2^ = 16.64%) experienced grade 3 or 4 IRRs (Additional file 1: Fig. S20). The results using Freeman-Tukey double arcsine transformation were similar to the results found. The results of the random-effects model were similar to those of the fixed-effects model, as shown in Additional file 1: Table S6.

## Discussion

This systematic review and meta-analysis of 30 studies evaluated the efficacy and safety of DARA IV-based therapies for the treatment of patients with AL amyloidosis. First, the results showed 66% of patients achieved ≥ VGPR. Subgroup analysis showed a slightly increased ≥ VGPR rate in patients treated with daratumumab-based triple regimens than mono or combined with only dexamethasone (71% vs. 63%). We also observed ≥ VGPR rates were similar for patients with primary or secondary AL amyloidosis (65% vs. 64%). Furthermore, rates of ≥ VGPR were higher in patients with newly diagnosed AL amyloidosis (84%) than in those with relapsed/refractory disease (67%). However, findings from the subgroup analysis should be interpreted with caution as additional studies are needed for confirmation [65].

In this study, the CR, VGPR and PR rates were 30%, 40% and 17%, respectively. In a prospective study including 915 patients with AL amyloidosis treated with bortezomib-based therapies, the CR rate was only 25%, and the cardiac and renal response rates were 32.5% and 15.4%, respectively [66]. A retrospective analysis of 25 patients with relapsed and refractory AL amyloidosis treated with DARA IV reported a hematologic response rate of 76%, with 36% of patients achieving CR and 24% achieving a very good partial response (VGPR) [38]. Moreover, 77% of patients achieved an overall response, 41% of patients with cardiac involvement exhibited a cardiac response, and 43% of patients with renal involvement showed a renal response.

This study revealed that 58% and 76% of patients reached one-year or longer PFS and OS, respectively, even though most included patients had relapsed/refractory AL amyloidosis. It is important to note that due to limited studies, only four studies in this review reported 1 year or longer progression-free survival rates with no more than 2 years follow-up. For comparison, in a study assessing the efficacy and safety of lenalidomide, melphalan and dexamethasone in AL amyloidosis patients with high rates of advanced cardiac involvement, the one-year survival rate was 58% [67]. This study suggested that treatment with DARA IV may improve survival for patients with AL amyloidosis.

Most of the IRRs were grade 1 or 2. 33% of patients experienced grade 1 or 2 IRRs and 3% of patients experienced grade 3 or 4 IRRs. The most common grade 3 or 4 adverse events reported were lymphocytopenia, heart failure, infection complications, pneumonia, fatigue, atrial fibrillation, neutropenia, and diarrhea. However, caution should be exercised when interpreting the results, as cardiac-related complications due to amyloid deposition are expected in AL amyloidosis and limitations of the meta-analysis. Besides, there were no new adverse events related to daratumumab. Overall, DARA IV therapy had an acceptable safety profile for patients with AL amyloidosis.

This study has several strengths. First, this study had good quality control. We conducted this study strictly according to the Cochrane and PRISMA standard. An information specialist performed the literature search. When necessary, a hand search was used to identify the reference lists of systematic reviews and conference abstracts. The screening and data extraction processes were performed independently by two reviewers and checked by a third independent assessor, ensuring the accuracy of the data. Second, to our knowledge, this study is the first comprehensive and the most current meta-analysis evaluating the efficacy and safety of DARA IV for the treatment of patients with AL amyloidosis. Third, we conducted subgroup analyses to reduce heterogeneity as much as possible.

There are nonetheless several limitations. Due to the language limitation, literature not in English and Chinese were excluded. All included studies were single arm studies or cohort studies, blinding and sample size calculation methods were not reported for most studies, which may either overestimate or underestimate the efficacy of DARA IV.

In conclusion, results of this meta-analysis suggests that DARA IV-based therapies are effective in generating hematologic and organ responses in both newly diagnosed and relapsed/refractory patients with AL amyloidosis. The safety profile is consistent with that has been previously documented for DARA IV in this population.

## Supplementary Information

**Additional file 1: Methods S1.** Search strategy. **Table** **S1**. Study quality based on (Before-After (Pre-Post)) outlined by National Institutes of Health (NIH). **Table S2.** Characteristics of studies include in this review. **Table S3.** The median time to first hematologic response. **Table S4.** The median time to best hematologic response. **Table S5.** Other adverse events in included studies. **Table S6.** Results of the random effects model in sensitivity analysis. **Fig. S1.** Meta-analysis forest plot of overall response rate. **Fig. S2.** Meta-analysis forest plot of complete remission. **Fig. S3.** Meta-analysis forest plot of very good partial response. **Fig. S4.** Meta-analysis forest plot of partial response. **Fig. S5.** Meta-analysis forest plot of cardiac response. **Fig. S6.** Meta-analysis forest plot of renal response. **Fig. S7.** Meta-analysis forest plot of ≥ VGPR-intervention. **Fig. S8.** Meta-analysis forest plot of ≥ VGPR-Mayo 2004. **Fig. S9.** Meta-analysis forest plot of ≥ VGPR-Mayo 2004 IIIA/B. **Fig. S10.** Meta-analysis forest plot of ≥ VGPR-primary or secondary. **Fig. S11.** Meta-analysis forest plot of ≥ VGPR-line of therapy. **Fig. S12.** Meta-analysis forest plot of PFS-1 year or longer. **Fig. S13.** Meta-analysis forest plot of OS-1 year or longer. **Fig. S14.** Meta-analysis forest plot of Infusion related reaction-grade-1–2. **Fig. S15.** Meta-analysis forest plot of Infusion related reaction-grade-3–4. **Fig. S16.** Meta-analysis forest plot of complete remission. **Fig. S17.** Meta-analysis forest plot of very good partial response. **Fig. S18.** Meta-analysis forest plot of partial response. **Fig. S19.** Meta-analysis forest plot of Renal response. **Fig. S20.** Meta-analysis forest plot of Infusion related reaction-grade-3–4.

## Data Availability

Not applicable.

## Abbreviations

DARA IV: Intravenous daratumumab
AL: Amyloid light-chain
FLCs: Free light chains
ASCT: Autologous stem cell transplantation
OS: Overall survival
CyBorD: Bortezomib-Cyclophosphamide-Dexamethasone
CR: Complete remission
DARA: Daratumumab
PRISMA: Preferred Reporting Items for Systematic Review and Meta-Analysis
INPLASY: International Platform of Registered Systematic Review and Meta-analysis Protocols
VGPR: Very good partial response
dFLC: Difference between involved and uninvolved free light chain
PR: Partial response
ORR: Overall response
NT-proBNP: N-terminal pro-brain natriuretic peptide
NYHA: New York Heart Association
PFS: Progression-free survival
AEs: Adverse events
IRRs: Infusion-related reactions
PICOS: Population, Intervention, Comparison, Outcome, Study design
eGFR: Epidermal growth factor receptor
RCTs: Randomized control trials
NRSIs: Non-randomized studies of interventions
NHLBI: National Heart, Lung, and Blood Institute
IQR: Interquartile range
RRs: Risk ratios
CIs: Confidence intervals
