# Supplementary material for: Efficacy and safety of intravenous daratumumab-based treatments for AL amyloidosis: a systematic review and meta-analysis
Source: Cancer Cell Int. 2022 Jul 4;22:222. doi: 10.1186/s12935-022-02635-6 (PMC9251945; doi:10.1186/s12935-022-02635-6)
Supplement: Supplementary file 1 — Additional file 1: Methods S1. Search strategy. Table S1. Study quality based on (Before-After (Pre-Post)) outlined by National Institutes of Health (NIH). Table S2. Characteristics of studies include in this review. Table S3. The median time to first hematologic response. Table S4. The median time to best hematologic response. Table S5. Other adverse events in included studies. Table S6. Results of the random effects model in sensitivity analysis. Fig. S1. Meta-analysis forest plot of overall response rate. Fig. S2. Meta-analysis forest plot of complete remission. Fig. S3. Meta-analysis forest plot of very good partial response. Fig. S4. Meta-analysis forest plot of partial response. Fig. S5. Meta-analysis forest plot of cardiac response. Fig. S6. Meta-analysis forest plot of renal response. Fig. S7. Meta-analysis forest plot of ≥ VGPR-intervention. Fig. S8. Meta-analysis forest plot of ≥ VGPR-Mayo 2004. Fig. S9. Meta-analysis forest plot of ≥ VGPR-Mayo 2004 IIIA/B. Fig. S10. Meta-analysis forest plot of ≥ VGPR-primary or secondary. Fig. S11. Meta-analysis forest plot of ≥ VGPR-line of therapy. Fig. S12. Meta-analysis forest plot of PFS-1 year or longer. Fig. S13. Meta-analysis forest plot of OS-1 year or longer. Fig. S14. Meta-analysis forest plot of Infusion related reaction-grade-1–2. Fig. S15. Meta-analysis forest plot of Infusion related reaction-grade-3–4. Fig. S16. Meta-analysis forest plot of complete remission. Fig. S17. Meta-analysis forest plot of very good partial response. Fig. S18. Meta-analysis forest plot of partial response. Fig. S19. Meta-analysis forest plot of Renal response. Fig. S20. Meta-analysis forest plot of Infusion related reaction-grade-3–4. [file 12935_2022_2635_MOESM1_ESM.docx]

**Methods S1: Search strategy**

**Table S1. Study quality based on (Before-After (Pre-Post)) outlined by National Institutes of Health (NIH)**

**Table S2. Characteristics of studies include in this review**

**Table S3. The median time to first hematologic response**

**Table S4. The median time to best hematologic response**

**Table S5. Other adverse events in included studies**

**Table S6. Results of the random effects model in sensitivity analysis**

**Figure S1. Meta-analysis forest plot of overall response rate**

**Figure S2. Meta-analysis forest plot of complete remission**

**Figure S3. Meta-analysis forest plot of very good partial response**

**Figure S4. Meta-analysis forest plot of partial response**

**Figure S5. Meta-analysis forest plot of cardiac response**

**Figure S6. Meta-analysis forest plot of renal response**

**Figure S7. Meta-analysis forest plot of ≥ VGPR-intervention**

**Figure S8. Meta-analysis forest plot of ≥ VGPR-Mayo 2004**

**Figure S9. Meta-analysis forest plot of ≥ VGPR-Mayo 2004 ⅢA/B**

**Figure S10. Meta-analysis forest plot of ≥ VGPR-primary or secondary**

**Figure S11. Meta-analysis forest plot of ≥ VGPR-line of therapy**

**Figure S12. Meta-analysis forest plot of PFS-1 year or longer**

**Figure S13. Meta-analysis forest plot of OS-1 year or longer**

**Figure S14. Meta-analysis forest plot of Infusion related reaction-grade-1-2**

**Figure S15. Meta-analysis forest plot of Infusion related reaction-grade-3-4**

**Figure S16. Meta-analysis forest plot of complete remission**

**Figure S17. Meta-analysis forest plot of very good partial response**

**Figure S18. Meta-analysis forest plot of partial response**

**Figure S19. Meta-analysis forest plot of Renal response**

**Figure S20. Meta-analysis forest plot of Infusion related reaction-grade-3-4**

**Methods S1: Search strategy**

1. EMBASEe
2. 'Daratumumab'/exp OR "humax CD38":ab,ti,kw OR darzalex:ab,ti,kw OR daratumumab:ab,ti,kw
3. 'amyloidosis'/exp OR (amyloidos* OR amyloid NEAR/2 tumor OR amyloid NEAR/2 tumour OR amyloido* OR paraamyloidos* OR Muckle NEAR/3 Wells NEAR/3 syndrome OR amyloid NEAR/2 neuropathy OR familial NEAR/2 Mediterranean NEAR/2 fever OR familial NEAR/3 amyloid NEAR/3 polyneuropathy OR Amyloid NEAR/3 Neuropathies OR Amyloid NEAR/3 Angiopathy):ab,ti,kw
4. #1 AND #2
5. PubMed
6. "daratumumab" [Supplementary Concept] OR Daratumumab[tw] OR "humax CD38"[tw] OR darzalex[tw]
7. "Amyloidosis"[Mesh] OR amyloidos*[tiab] OR amyloid tumor[tiab] OR amyloid tumour[tiab] OR amyloido*[tiab] OR paraamyloidos*[tiab] OR Muckle Wells syndrome[tiab] OR amyloid neuropathy[tiab] OR familial Mediterranean fever[tiab] OR familial amyloid polyneuropathy[tiab] OR Amyloid Neuropathies[tiab] OR Amyloid Angiopathy[tiab]
8. #1 AND #2
9. Web of science
10. TS=(Daratumumab OR "humax CD38" OR darzalex)
11. TS=(amyloidos* OR amyloid NEAR/1 tumor OR amyloid NEAR/1 tumour OR amyloido* OR paraamyloidos* OR Muckle NEAR/1 Wells NEAR/1 syndrome OR amyloid NEAR/1 neuropathy OR familial NEAR/1 Mediterranean NEAR/1 fever OR familial NEAR/1 amyloid NEAR/1 polyneuropathy OR Amyloid NEAR/3 Neuropathies OR Amyloid NEAR/3 Angiopathy)
12. #1 AND #2
13. Cochrane library

#1 Daratumumab OR "humax CD38" OR darzalex

#2 MeSH descriptor: [Amyloidosis] explode all trees

#3 (amyloidos* OR amyloid NEAR/1 tumor OR amyloid NEAR/1 tumour OR amyloido* OR paraamyloidos* OR Muckle NEAR/1 Wells NEAR/1 syndrome OR amyloid NEAR/1 neuropathy OR familial NEAR/1 Mediterranean NEAR/1 fever OR familial NEAR/1 amyloid NEAR/1 polyneuropathy OR Amyloid NEAR/3 Neuropathies OR Amyloid NEAR/3 Angiopathy):ti,ab,kw

#4 #2 or #3

#5 #1 and #4

**Table S1. Study quality based on (Before-After (Pre-Post)) outlined by National Institutes of Health (NIH)**

| Study ID |  | Abeykoon 2018 | Chung 2020 | Cohen 2020 | Dima 2020 | Fazio 2019 | Godara 2019 | Gounot 2020 | Jeryczynski 2021 | Kastritis 2019 | Kaufman 2017 |
| --- | --- | --- | --- | --- | --- | --- | --- | --- | --- | --- | --- |
| Question/objective | ·Study question or objective clearly stated | Yes | Yes | Yes | Yes | Yes | Yes | Yes | Yes | Yes | Yes |
| Participants | ·Eligibility/selection criteria for the study population prespecified and clearly described | Yes | Yes | Yes | Yes | No | Yes | Yes | Yes | No | Yes |
|  | ·Participants in the study representative of those who would be eligible for the test/service/intervention in the general or clinical population of interest | Yes | Yes | Yes | NR | Yes | Yes | Yes | NR | Yes | Yes |
|  | ·All eligible participants that met the prespecified entry criteria enrolled | Yes | Yes | Yes | NR | NR | Yes | Yes | NR | Yes | Yes |
|  | ·Sample size sufficiently large to provide confidence in the findings | CD | CD | CD | CD | CD | CD | CD | CD | CD | CD |
|  | ·Test/service/intervention clearly described and delivered consistently across the study population | Yes | Yes | Yes | NA | NA | Yes | Yes | Yes | Yes | Yes |
| Outcomes | ·Outcome measures prespecified, clearly defined, valid, reliable, and assessed consistently across all study participants | Yes | Yes | Yes | CD | NA | Yes | Yes | Yes | Yes | No |
|  | ·People assessing the outcomes blinded to the participants' exposures/interventions | NR | NR | NR | NR | NR | NR | NR | NR | NR | NR |
|  | ·Loss to follow-up after baseline 20% or less, those lost to follow-up accounted for in the analysis | Yes | Yes | No | Yes | Yes | Yes | No | Yes | Yes | No |
| Statistical analysis | ·Statistical methods examine changes in outcome measures from before to after the intervention, statistical tests done that provided p values for the pre-to-post changes | No | No | No | No | No | No | No | No | No | No |
|  | ·Outcome measures of interest taken multiple times before the intervention and multiple times after the intervention (i.e., did they use an interrupted time-series design) | No | No | No | No | No | Yes | No | No | Yes | No |
|  | ·Intervention was conducted at a group level (e.g., a whole hospital, a community, etc.) did the statistical analysis take into account the use of individual-level data to determine effects at the group level | NA | NA | NA | NA | NA | NA | NA | NA | NA | NA |

| **Study ID** |  | Kennedy  2020 | Khouri 2018 | Kimmich 2020 | Kimmich 2021 | Kleman 2019 | Lecumberri 2020 | Lee  2018 | Lee  2019 | Milani 2020 | Ozga 2019 |
| --- | --- | --- | --- | --- | --- | --- | --- | --- | --- | --- | --- |
| **Question/objective** | ·Study question or objective clearly stated | Yes | Yes | Yes | Yes | Yes | Yes | Yes | Yes | Yes | Yes |
| **Participants** | ·Eligibility/selection criteria for the study population prespecified and clearly described | Yes | Yes | Yes | No | Yes | Yes | Yes | Yes | Yes | Yes |
|  | ·Participants in the study representative of those who would be eligible for the test/service/intervention in the general or clinical population of interest | NR | Yes | Yes | NR | NR | Yes | NR | NR | Yes | NR |
|  | ·All eligible participants that met the prespecified entry criteria enrolled | NR | Yes | Yes | NR | NR | NR | NR | NR | Yes | NR |
|  | ·Sample size sufficiently large to provide confidence in the findings | CD | CD | CD | CD | CD | CD | CD | CD | CD | CD |
|  | ·Test/service/intervention clearly described and delivered consistently across the study population | NA | Yes | Yes | Yes | NA | No | Yes | Yes | Yes | NA |
| **Outcomes** | ·Outcome measures prespecified, clearly defined, valid, reliable, and assessed consistently across all study participants | CD | Yes | Yes | CD | CD | No | Yes | Yes | Yes | CD |
|  | ·People assessing the outcomes blinded to the participants' exposures/interventions | NR | NR | NR | NR | NR | NR | NR | NR | NR | NR |
|  | ·Loss to follow-up after baseline 20% or less, those lost to follow-up accounted for in the analysis | Yes | Yes | No | Yes | Yes | Yes | Yes | Yes | Yes | Yes |
| **Statistical analysis** | ·Statistical methods examine changes in outcome measures from before to after the intervention, statistical tests done that provided p values for the pre-to-post changes | No | No | No | No | Yes | No | No | No | No | No |
|  | ·Outcome measures of interest taken multiple times before the intervention and multiple times after the intervention (i.e., did they use an interrupted time-series design) | No | No | Yes | No | Yes | No | No | No | No | No |
|  | ·Intervention was conducted at a group level (e.g., a whole hospital, a community, etc.) did the statistical analysis take into account the use of individual-level data to determine effects at the group level | NA | NA | NA | NA | NA | NA | NA | NA | NA | NA |

| **Study ID** |  | Pick 2018 | Rahel 2019 | Ratermann 2019 | Riva 2019 | Rosko 2019 | Roussel 2020 | Sanchorawala 2020 | Shragai 2020 | Sidiqi 2019 | Wyngaert 2019 |
| --- | --- | --- | --- | --- | --- | --- | --- | --- | --- | --- | --- |
| **Question/objective** | ·Study question or objective clearly stated | Yes | Yes | Yes | Yes | Yes | Yes | Yes | Yes | Yes | Yes |
| **Participants** | ·Eligibility/selection criteria for the study population prespecified and clearly described | Yes | Yes | Yes | Yes | Yes | Yes | Yes | Yes | Yes | Yes |
|  | ·Participants in the study representative of those who would be eligible for the test/service/intervention in the general or clinical population of interest | Yes | Yes | NR | NR | NR | Yes | Yes | Yes | NR | Yes |
|  | ·All eligible participants that met the prespecified entry criteria enrolled | Yes | Yes | NR | NR | NR | Yes | Yes | Yes | NR | Yes |
|  | ·Sample size sufficiently large to provide confidence in the findings | CD | CD | CD | CD | CD | Yes | CD | CD | CD | CD |
|  | ·Test/service/intervention clearly described and delivered consistently across the study population | No | Yes | NA | Yes | Yes | Yes | Yes | Yes | NA | Yes |
| **Outcomes** | ·Outcome measures prespecified, clearly defined, valid, reliable, and assessed consistently across all study participants | Yes | Yes | CD | Yes | No | Yes | Yes | Yes | CD | Yes |
|  | ·People assessing the outcomes blinded to the participants' exposures/interventions | NR | NR | NR | NR | NR | NR | NR | NR | NR | NR |
|  | ·Loss to follow-up after baseline 20% or less, those lost to follow-up accounted for in the analysis | Yes | Yes | Yes | Yes | Yes | Yes | Yes | Yes | Yes | Yes |
| **Statistical analysis** | ·Statistical methods examine changes in outcome measures from before to after the intervention, statistical tests done that provided p values for the pre-to-post changes | No | No | No | No | No | No | No | No | No | No |
|  | ·Outcome measures of interest taken multiple times before the intervention and multiple times after the intervention (i.e., did they use an interrupted time-series design) | No | No | No | No | No | No | No | No | No | No |
|  | ·Intervention was conducted at a group level (e.g., a whole hospital, a community, etc.) did the statistical analysis take into account the use of individual-level data to determine effects at the group level | NA | NA | NA | NA | NA | NA | NA | NA | NA | NA |

Notes:

CD = cannot determine; NA = not applicable; NR = not reported

**Table S2. Characteristics of studies include in this review**

| Ref nubmer | Study ID | Country | Centre | Newly diagnosed or relapsed | Total sample size, n | Gender, n | Mayo Stage 2012 n | Type of AL,  n | Age  (years)  Media (range) | Involved organs,  n | eGFR  (mL/min/ m^2^) | dFLC  (mg/L)  Media (range) | Lines of therapy Media (range) | Intervention | n of infusions  Median  (range) | Outcome |
| --- | --- | --- | --- | --- | --- | --- | --- | --- | --- | --- | --- | --- | --- | --- | --- | --- |
| 22 | Abeykoon 2018 | USA | Single | Relapsed and/or refractory | Arm 1: 22 | NR | Ⅰ: 2  Ⅱ: 7  Ⅲ: 7  Ⅳ: 4 | NR | NR | Heart: 14  Kidney: 11  Liver: 2 | NR | 70  (10–700) | 4 (2–6) | Dara ± dex | 8.0 (2–24) | HR  OR  SUR  AE |
|  |  |  |  |  | Arm 2: 22 | NR | Ⅰ: 1  Ⅱ: 12  Ⅲ: 5  Ⅳ: 2 | NR | NR | Heart: 13  Kidney: 11  Liver: 1 | NR | 101  (6–3010) | 4 (2–9) | Mixed^*^ | 8.0 (2–24) | HR  OR  SUR  AE |
| 22–24 | Chung 2020 | USA | Single | NR | 72 | Male: 44  Female: 28 | 2004  Ⅰ: 10  Ⅱ: 37  ⅢA: 12  ⅢB: 5 | λ: 54  k: 18 | 67  IQR  (60–72) | Heart: 57  Kidney: 47  Liver: 5  Heart and kidney: 35 | NR | 34.5  IQR  (18–107) | 3  IQR (2–4) | Dara ± dex | 24 weeks + a maintenance dose Q4W | HR  OR  SUR  AE |
| 25–26 | Cohen 2020 | UK | NR | Relapsed/refractory | 53 | Male: 34  Female: 19 | 2004  Ⅰ: 11  Ⅱ: 19  ⅢA: 18  ⅢB: 5 | λ: 36  k: NR | 68  (42–85) | Heart: 39  Kidney: 30  Liver: 14 | 51.5 (15–90) | 78.9  (0.3–4897) | 4 (2–5) | Dara ± dex | NR | HR  OR  SUR  AE |
| 27 | Dima 2020 | USA | Single | NR | 40 | Male: 22  Female: 18 | NR | NR | 66  (35–80) | Heart: 30  Kidney: 20  Other: 12 | NR | NR | 1: 10  2–3: 15  >3: 15 | Dara ± dex + McAb | NR | HR  OR  SUR  AE |
| 28 | Fazio 2019 | Italy | Single | Relapsed/refractory | 46 | Male: 27  Female: 19 | NR | NR | 62  (34–79) | Heart: 33  Kidney: 31 | NR | NR | 4 (2–10) | Mixed | NR | HR  OR  SUR  AE |
| 29–30 | Godara 2019 | USA | Single | N + R | Arm 1: 9 | Male: 4  Female: 5 | 2004  Ⅱ: 3  Ⅲ: 5 | λ: 8  k: 1 | 68  (52–75) | Heart: 8  Kidney: 4 | NR | NR | 4 (2–6) | Dara ± dex + McAb | 258 (47–637) days | HR  OR  SUR  AE |
|  |  |  |  |  | Arm 2: 10 | Male: 8  Female: 2 | 2004  Ⅰ: 0  Ⅱ: 10  Ⅲ: 0 | λ: 7  k: 3 | 66.5  (54–74) | Heart: 7  Kidney: 8 | NR | NR | 2 (2–4) | Dara ± dex | NR | HR  OR  SUR  AE |
| 31–32 | Gounot 2020 | France | Single | N + R | 25 | Male: 18  Female: 7 | 2004  Ⅰ: 0  Ⅱ: 0  Ⅲ: 25 | λ: 20  k: NR | 65  (52–83) | Heart: 25  Kidney: 12  Liver: 2  Pulmonary: 1 | 34.8  (10.5–76.5) | 108.6  (37.6–1100.7) | 2 (1–5) | Mixed | 4  (0.25–11) | HR  OR  SUR  AE |
| 34–35 | Jeryczynski 2021 | Austria | Single | Newly diagnose | Arm 1: 7 | Male: 5  Female: 2 | Ⅰ: 1  Ⅱ: 1  Ⅲ: 1  Ⅳ: 4 | λ: 7  k: 0 | 71.8  (49–80.7) | Heart: 6  Kidney: 4  Other: 1 | NR | 276.9  (100.6–2924.4) | 1 | Dara ± dex | 16 (3–21) | HR  OR |
|  |  |  |  |  | Arm 2: 7 | Male: 4  Female: 3 | Ⅰ: 1  Ⅱ: 1  Ⅲ: 2  Ⅳ: 3 | λ: 5  k: 2 | 62.7  (47.8–80.3) | Heart: 5  Kidney: 5  Other: 1 | NR | 223.5  (21–954.6) | 1 | Mixed | 16 (3–21) | HR  OR |
| 36 | Kastritis 2019 | NR | NR | NR | 23 | NR | NR | λ: 19  k: NR | NR | NR | NR | NR | NR | Dara ± dex | 4 | HR |
| 37–38 | Kaufman 2017 | USA | Single | Refractory diagnose | 25 | Male: 17  Female: 8 | 2004  Ⅰ: 4  Ⅱ: 11  Ⅲ: 10 | NR | 66  (50–76) | Heart: 18  Kidney: 17 | 47(10–90) | 74  (4–280) | 4 (2–6) | Dara ± dex | 12 (3–35) | HR  SUR  AE |
| 39 | Kennedy 2020 | USA | Single | Newly diagnose | 14 | Male: 7  Female: 7 | 2004  I: 1  Ⅱ: 2  Ⅲ: 11 | λ: 7  k: NR | 69  (45–87) | Heart: 14  Kidney: 14 | NR | NR | 1 | Mixed | 5 (1–14) | HR  OR  AE |
| 40–41 | Khouri 2018 | USA | Multi | Relapsed | 20 | Male: 10  Female: 10 | NR | NR | 67  (40–88) | Heart: 10  Kidney: 7  Tongue: 4 | NR | 88.7  (13.8–680) | 4 (2–11) | Dara ± dex | 17 (6–34) | HR  SUR  AE |
| 42–43 | Kimmich 2020 | Germany | Multi | N + R | Arm 1: 106 | Male: 73  Female: 33 | NR | λ: 90  k: NR | 65  (36–81) | Heart: 88  Kidney: 67  Liver: 15 | 51.5  (12–126) | 236  (22–9600) | 3 (2–8) | Dara ± dex | 14 (2–35) | HR  OR  SUR  AE |
|  |  |  |  |  | Arm 2: 62 | Male: 33  Female: 29 | NR | λ: 47  k: NR | 60  (38–79) | Heart: 54  Kidney: 40  Liver: 15 | 53.5  (11– 106) | 247  (0–2237) | 2 (2–8) | DVd | 14 (1–29) | HR  OR  SUR  AE |
| 44 | Kimmich 2021 | Germany | Multi | NR | 44 | Male: 31  Female: 13 | NR | NR | 62  (50–80) | Heart: 34  Kidney: 26  Liver: 3 | NR | 102  (0–3394) | 2: 27  3: 11  4: 6 | DRd | 17 (2–38) | HR  OR  SUR  AE |
| 45 | Kleman 2019 | USA | Multi | N + R | 42 | Male: 26  Female: 16 | Ⅰ–Ⅱ: 18  Ⅲ–Ⅳ: 24 | NR | 67  (42–91) | Heart: 32  Kidney: 18 | NR | NR | 2 (1–8) | Mixed | 6.8 (0–22) months | SUR |
| 16 | Lecumberri 2020 | Spain | Multi | Relapsed/refractory | 38 | Male: 23  Female: 15 | Ⅰ: 6  Ⅱ: 9  Ⅲ: 12  Ⅳ: 10 | NR | NR | Heart: 29  Kidney: 28 | NR | 150  (6–2389) | 3 (2–9) | Mixed | 18 (1–38) | HR  OR  SUR  AE |
| 46–47 | Lee 2019 | USA | NR | Relapsed/refractory | 17 | NR | NR | NR | NR | Heart: 11  Kidney: 12 | 34  (0–98)  n = 12* | NR | 4 (2–6) | Dara ± dex | NR | HR  OR  SUR |
| 48 | Lee 2018 | USA | NR | Relapsed/refractory | 10 | Male: 8  Female: 2 | NR | NR | 67  (38–75) | Cardiad/Renal  GI/Lung  and/or bone marrow | NR | NR | 2 | Mixed | 5 (1–9) | HR  OR  AE |
| 49–50 | Milani 2020 | Italy | Multi | Relapsed/refractory | 72 | Male: 45  Female: 27 | NR | NR | 62  (37–79) | Heart: 49  Kidney: 49  Liver: 3 | NR | 118  IQR  (55–1099) | 3 (2–10) | Mixed | 28  (Living patients) | HR  OR  SUR  AE |
| 51 | Ozga 2019 | USA | Single | NR | 20 | NR | NR | NR | NR | NR | NR | NR | NR | Dara ± dex | NR | HR  SUR |
| 52 | Pick 2018 | Israel | Single | Refractory diagnose | 4 | Male: 2  Female: 2 | NR | λ: 2  k: 2 | 58  (51–75) | NR | NR | NR | 6 (2–10) | Mixed | NR | HR  AE |
| 17 | Rahel 2019 | Swiss | Multi | Relapsed/refractory | 10 | Male: 8  Female: 2 | Ⅰ: 0  Ⅱ: 3  Ⅲ: 5  Ⅳ: 2 | NR | 62.3  (39.7–73.2) | Heart: 10  Kidney: 6 | 73  (25–108) | 191  (40–1382) | 4 (2–6) | Dara ± dex | 18  (8–23) | HR  OR  AE |
| 53 | Ratermann 2019 | USA | Single | Newly diagnose | Arm 1: 2 | NR | NR | NR | NR | NR | NR | NR | 1 | DCd | NR | HR |
|  |  |  |  | Relapsed/refractory  Relapsed/refractory | Arm 2: 2  Arm 3: 1 | NR  NR | NR  NR | NR  NR | NR  NR | NR  NR | NR  NR | NR  NR | NR  NR | DRd  Dara ± dex | NR  NR | HR  HR |
| 54 | Riva 2019 | Italy | Single | Refractory diagnose | 10 | Male: 6  Female: 4 | NR | λ: 9  k: 1 | 63.2  (53–77) | Heart: 2  Kidney: 1  Heart and renal: 6 | <50: n = 4 | 4640  (280–23530) | ≥ 2 | Dara ± dex | NR | HR  AE |
| 55 | Rosko 2019 | USA | NR | NR | 27 | Male: 15  Female: 12 | NR | NR | 72 | Heart: 17  Kidney: 14 | NR | NR | NR | Dara ± dex | NR | AE |
| 2, 33, 56–57 | Roussel 2020 | 14 centers# and Italy | Multi | Refractory diagnose | 40 | Male: 25  Female: 15 | 2004  Ⅰ: 11  Ⅱ: 10  ⅢA: 19 | λ: 30  k: NR | 69  (45–83) | Heart: 24  Kidney: 26  Liver: 4 | NR | 164  IQR  (112–334) | 4  IQR (2.75–4) | Dara ± dex | ≥ 6 cycles  82.5% patients | HR  OR  SUR  AE |
| 58–60 | Sanchorawala 2020 | USA | Single | Refractory diagnose | 22 | Male: 16  Female: 6 | NR | λ: 17  k: NR | 63  (42–83) | Heart: 14  Kidney: 15 | 58  (20–112) | 80.7  (2.9–854) | 3 (2–8) | Dara ± dex | 31 (7–34) | HR  OR  SUR  AE |
| 61 | Shragai 2020 | Israel | Multi | Relapsed/refractory | 49 | Male: 30  Female: 19 | NR | λ: 29  k: 20 | 62  (47–90) | Heart: 35  Kidney: 28  Liver: 7  Other: 6 | NR | 134  IQR  (68–279) | 2 (2–7) | Mixed | 8 (1–28) | HR  OR  SUR  AE |
| 62 | Sidiqi 2019 | USA | Single | Refractory diagnose | 9 | Male: 6  Female: 3 | Ⅰ: 3  Ⅱ: 3  Ⅲ: 1  Ⅳ: 1 | NR | 65  (51–73) | NR | NR | NR | ≥ 2 | Unclear  (Dara based) | 10.2 month | HR  SUR |
| 63–64 | Wyngaert 2019 | France | Multi | Relapsed/refractory | 15 | Male: 10  Female: 5 | NR | λ: NR  k: 7 | 60  IQR  (47–67) | Heart: 10  Kidney: 9  Liver: 5  Spleen 2  UAT: 4  Lung: 2  Muscle: 1 | 37  IQR  (35–48) | 121  IQR  (69–967) | 3 (2–6) | Dara ± dex | 12  (0.25–19) | HR  OR  SUR  AE |

Notes:

AE = adverse event; Dara ± dex = Daratumumab ± Dexamethasone, Dara + McAb = Daratumumab + NEOD001, DVd = Daratumumab + Bortezomib + Dexamethasone, DRd = Daratumumab + Lenalidomide + Dexamethasone, DCd = Daratumumab + Cyclophosphamide + Dexamethasone, eGFR = estimated glomerular filtration rate, HR = hematologic response, IQR = interquartile range, N + R = newly diagnosed and relapsed, NR = not reported, OR = organ response, SUR = survival

^*^Mixed Daratumumab–based treatments indicate there were more than one kind of daratumumab–based treatment in the study (daratumumab may be combined with dexamethasone, bortezomib, lenalidomide, pomalidomide, cyclophosphamide, ixazomid, carfilzomib, etc.)

**Table S3. The median time to first hematologic response**

| Study ID | Intervention | Median (days) | Range or 95% CI | Total (n) |
| --- | --- | --- | --- | --- |
| Abeykoon 2018 | Dara ± dex | 78 | 95% CI (51, 186) | 14 |
| Cohen 2020 | Dara ± dex | 30 | Range (30－180) | 50 |
| Godara 2019 | Dara ± dex | 75 | Range (28－250) | 10 |
| Khouri 2018 | Dara ± dex | 28 | Not reported | 15 |
| Kimmich 2020 | Dara ± dex | 49 | Range (5－287) | 32 |
| Roussel 2020 | Dara ± dex | 7 | Not reported | 40 |
| Sanchorawala 2020 | Dara ± dex | 53 | Not reported | 22 |
| Godara 2019 | Dara + McAb | 33 | Range (19－161) | 9 |
| Chung 2020 | Dara ± dex | 30 | Not reported | 52 |
| Lecumberri 2020 | Dara ± dex | 14 | Range (7－84) | 36 |
| Rahel 2019 | Dara ± dex | 14 | Range (7－28) | 10 |
| Abeykoon 2018 | Mixed^*^ | 57 | 95% CI (27, 102) | 16 |
| Dima 2020 | Mixed | 45 | 95% CI (30, 60) | 40 |
| Gounot 2020 | Mixed | 45 | Range (1－4) | 23 |
| Jeryczynski 2021 | Mixed | 42 | Rang (6－249) | 14 |
| Kennedy 2020 | Mixed | 11 | Range (7－61) | 14 |
| Wyngaert 2019 | Dara ± dex | 28 (mean, days) | Not reported | 14 |

Notes:

Dara ± dex = Daratumumab ± Dexamethasone, Dara + McAb = Daratumumab + NEOD001

^*^Mixed Daratumumab-based treatments indicate there were more than one kind of daratumumab-based treatment in the study (daratumumab may be combined with dexamethasone, bortezomib, lenalidomide, pomalidomide, cyclophosphamide, ixazomid, carfilzomib, etc.)

**Table S4. The median time to best hematologic response**

| Study ID | Intervention | Median (days) | Range or 95% CI | Total (n) |
| --- | --- | --- | --- | --- |
| Abeykoon 2018 | Dara ± dex | 186 | 95% CI (132, 267) | 14 |
| Lee 2019 | Dara ± dex | 81 | Not reported | 17 |
| Sanchorawala 2020 | Dara ± dex | 90 | Range (90－360) | 22 |
| Wyngaert 2019 | Dara ± dex | 70 | Range (28－350) | 14 |
| Kaufman 2017 | Dara ± dex | 30 | Range (7－188) | 25 |
| Lecumberri 2020 | Dara ± dex | 60 | Range (30－330) | 36 |
| Rahel 2019 | Dara ± dex | 73 | Range (7－301) | 10 |
| Abeykoon 2018 | Mixed^*^ | 171 | 95% CI (69, 366) | 16 |
| Gounot 2020 | Mixed | 75 | Range (30－150) | 23 |
| Jeryczynski 2021 | Mixed | 336 | Range (15－630) | 14 |
| Kennedy 2020 | Mixed | 46 | Range (9.8－168) | 14 |
| Shragai 2020 | Mixed | 60 | Range (15－300) | 48 |

Notes:

Dara ± dex = Daratumumab ± Dexamethasone

^*^Mixed Daratumumab-based treatments indicate there were more than one kind of daratumumab-based treatment in the study (daratumumab may be combined with dexamethasone, bortezomib, lenalidomide, pomalidomide, cyclophosphamide, ixazomid, carfilzomib, etc.)

**Table S5. Other adverse events in included studies**

| Adverse event | Study ID | Intervention | Event | Total (n) | Percentage (%) |
| --- | --- | --- | --- | --- | --- |
| Cardiovascular toxicity | Shragai 2020 | Mixed^*^ | 4 | 48 | 8.3 |
| Dyspnea | Shragai 2020 | Mixed | 4 | 48 | 8.3 |
| Peripheral neuropathy | Shragai 2020 | Mixed | 4 | 48 | 8.3 |
| Large bowel perforation related to colitis | Kimmich 2021 | DRd | 3 | 44 | 6.8 |
| Cutaneous rash | Roussel 2020 | Dara ± dex | 2 | 40 | 5.0 |
| Gastrointestinal bleeding | Sanchorawala 2020 | Dara ± dex | 1 | 22 | 4.5 |
| Increased creatinine | Sanchorawala 2020 | Dara ± dex | 1 | 22 | 4.5 |
| Skin abscesses | Kimmich 2020 | DRd | 2 | 44 | 4.5 |
| Hypersensitivity reaction | Shragai 2020 | Mixed | 2 | 48 | 4.2 |
| Renal toxicity | Shragai 2020 | Mixed | 2 | 48 | 4.2 |
| Spontaneous pneumothorax | Kaufman 2017 | Dara ± dex | 1 | 25 | 4.0 |
| Decompensated heart failure | Kaufman 2017 | Dara ± dex | 1 | 25 | 4.0 |
| Fall | Cohen 2020 | Dara ± dex | 2 | 53 | 3.8 |
| Fluid overload | Cohen 2020 | Dara ± dex | 2 | 53 | 3.8 |
| Other | Kimmich 2020 | Dara ± dex, DVd | 5 | 168 | 3.0 |
| Acute renal failure and hypokalaemia | Lecumberri 2020 | Mixed | 1 | 38 | 2.6 |
| Leucopenia | Roussel 2020 | Dara ± dex | 1 | 40 | 2.5 |
| Orthostatic hypotension | Roussel 2020 | Dara ± dex | 1 | 40 | 2.5 |
| Septic shock-lethal infection | Dima 2020 | Mixed | 1 | 40 | 2.5 |
| Anaemia | Abeykoon 2018 | Mixed | 1 | 44 | 2.3 |
| Hematologic toxicity | Kimmich 2021 | DRd | 1 | 44 | 2.3 |
| Nausea/vomiting | Shragai 2020 | Mixed | 1 | 48 | 2.1 |
| Thrombocytopenia | Cohen 2020 | Dara ± dex | 0 | 53 | 0.0 |
|  | Kimmich 2020 | Dara ± dex, DVd | 1 | 149 | 0.7 |
|  | Milani 2020 | Mixed | 2 | 72 | 2.8 |
| Blurred vision | Cohen 2020 | Dara ± dex | 0 | 53 | 0.0 |
| Cellulitis | Dima 2020 | Mixed | 0 | 40 | 0.0 |
| Hypertension | Cohen 2020 | Dara ± dex | 0 | 53 | 0.0 |
| Insomnia | Cohen 2020 | Dara ± dex | 0 | 53 | 0.0 |
| Nausea | Cohen 2020 | Dara ± dex | 0 | 53 | 0.0 |
| Polyneuropathy | Kimmich 2020 | Dara ± dex, DVd | 0 | 168 | 0.0 |
| Viral gastroenteritis | Dima 2020 | Mixed | 0 | 40 | 0.0 |

Note:

Dara ± dex = Daratumumab ± Dexamethasone, DVd = Daratumumab + Bortezomib + Dexamethasone, DRd = Daratumumab + Lenalidomide + Dexamethasone

^*^Mixed Daratumumab-based treatments indicate there were more than one kind of daratumumab-based treatment in the study (daratumumab may be combined with dexamethasone, bortezomib, lenalidomide, pomalidomide, cyclophosphamide, ixazomid, carfilzomib, etc.)

**Table S6. Results of the random effects model in sensitivity analysis**

| Outcome or Subgroup | Studies | Patients (n) | Effect Estimate | I^2^ |
| --- | --- | --- | --- | --- |
| Overall response rate | 26 | 786 | 0.81 [0.76, 0.85] | 45% |
| Partial response | 19 | 544 | 0.17 [0.12, 0.22] | 47% |
| ≥ VGPR-intervention |  |  |  |  |
| Dara +/- dexamethasone | 16 | 387 | 0.66 [0.59, 0.73] | 39% |
| Triple regimens | 3 | 89 | 0.72 [0.56, 0.84] | 35% |
| ≥ VGPR-Mayo 2004 |  |  |  |  |
| Mayo Ⅰ | 1 | 10 | 0.40 [0.12, 0.74] | — |
| Mayo Ⅱ | 3 | 38 | 0.73 [0.57, 0.85] | 0% |
| Mayo Ⅲ | 3 | 54 | 0.70 [0.66, 1.00] | 48% |
| PFS-1 year or longer | 3 | 126 | 0.58 [0.45, 0.69] | 46% |
| OS-1 year or longer | 11 | 534 | 0.78 [0.73, 0.82] | 31% |
| Infusion related reaction-grade -3-4 | 10 | 437 | 0.03 [0.02, 0.06] | 0% |


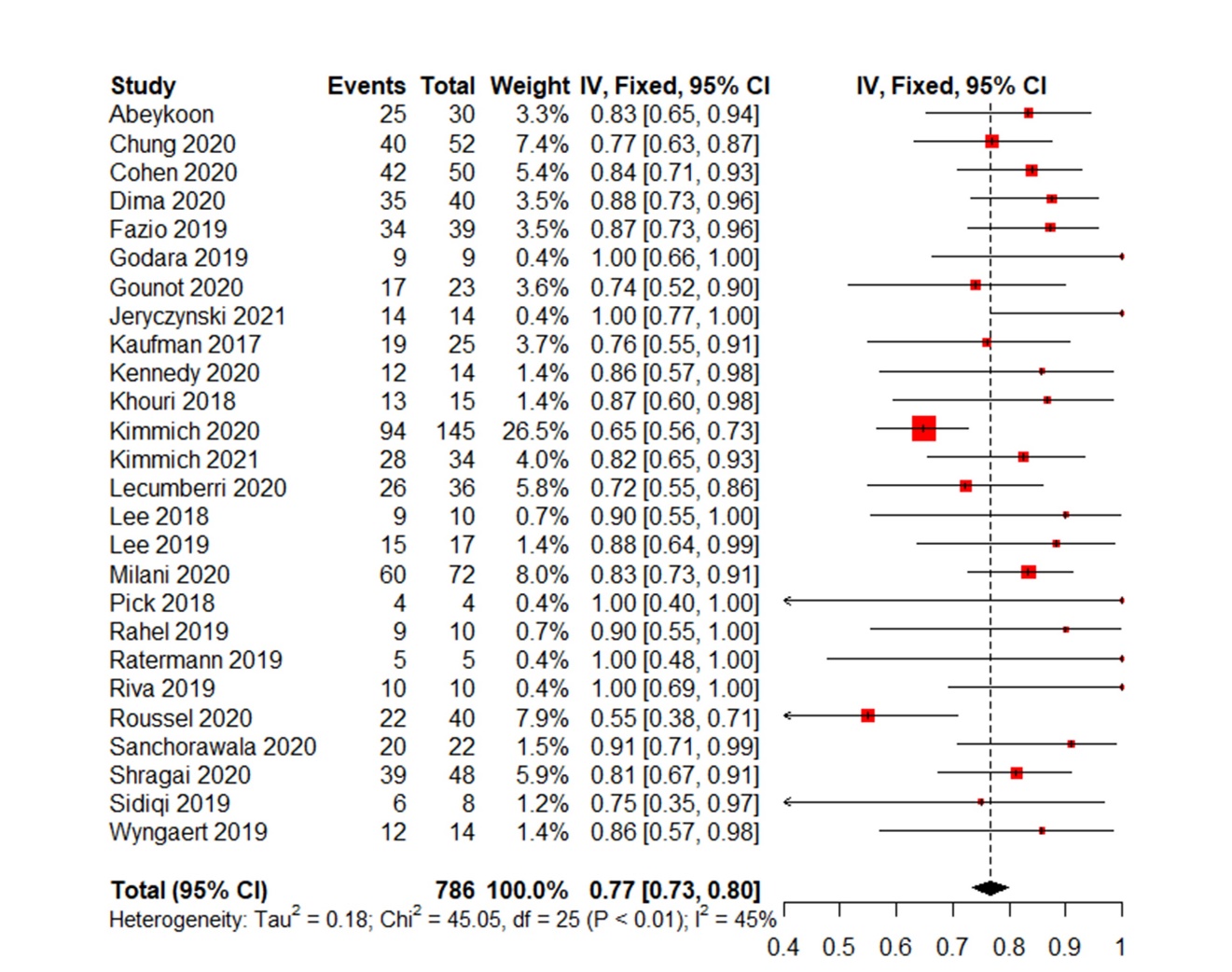
**Figure S1. Meta-analysis forest plot of overall response rate**


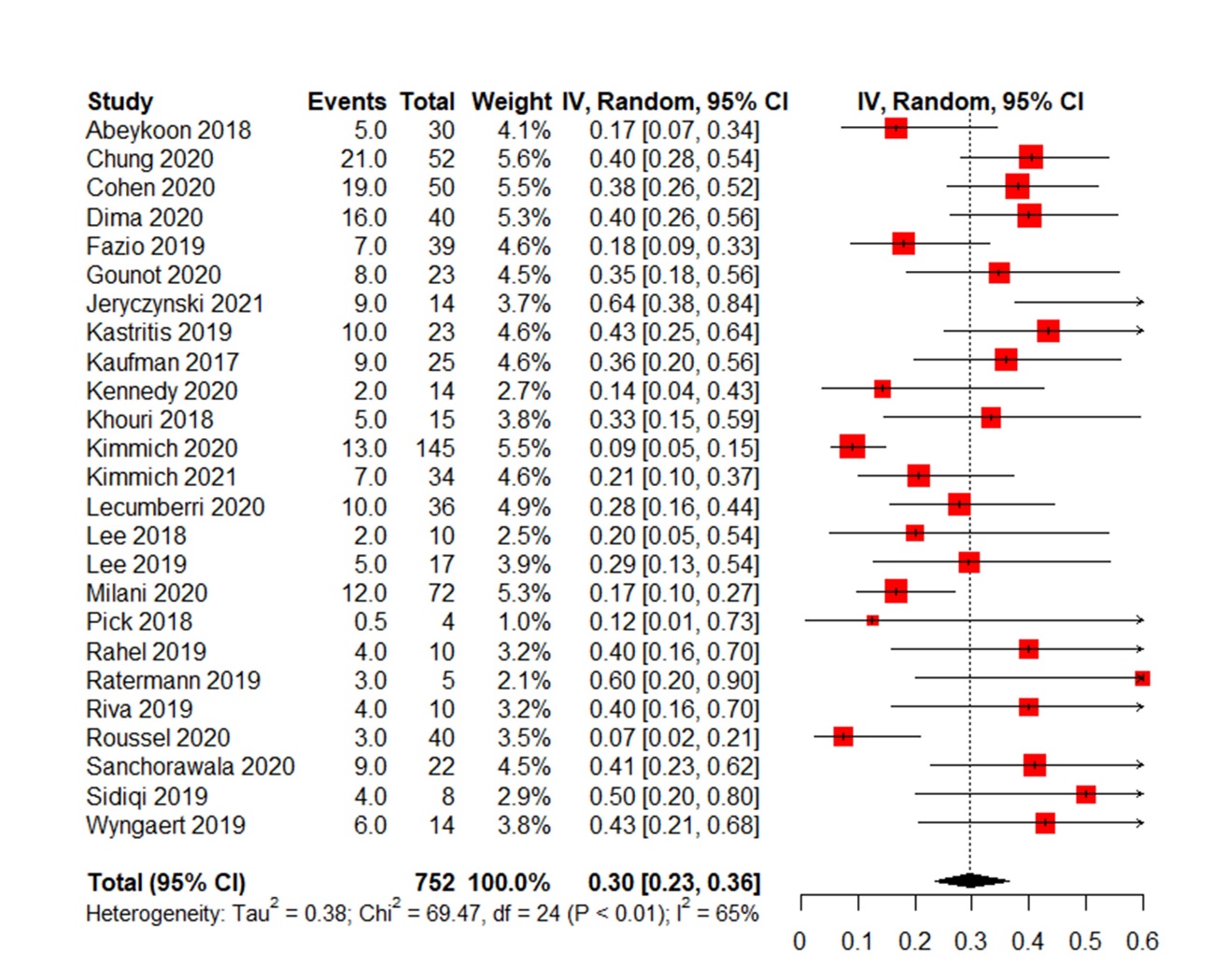
**Figure S2. Meta-analysis forest plot of complete remission**


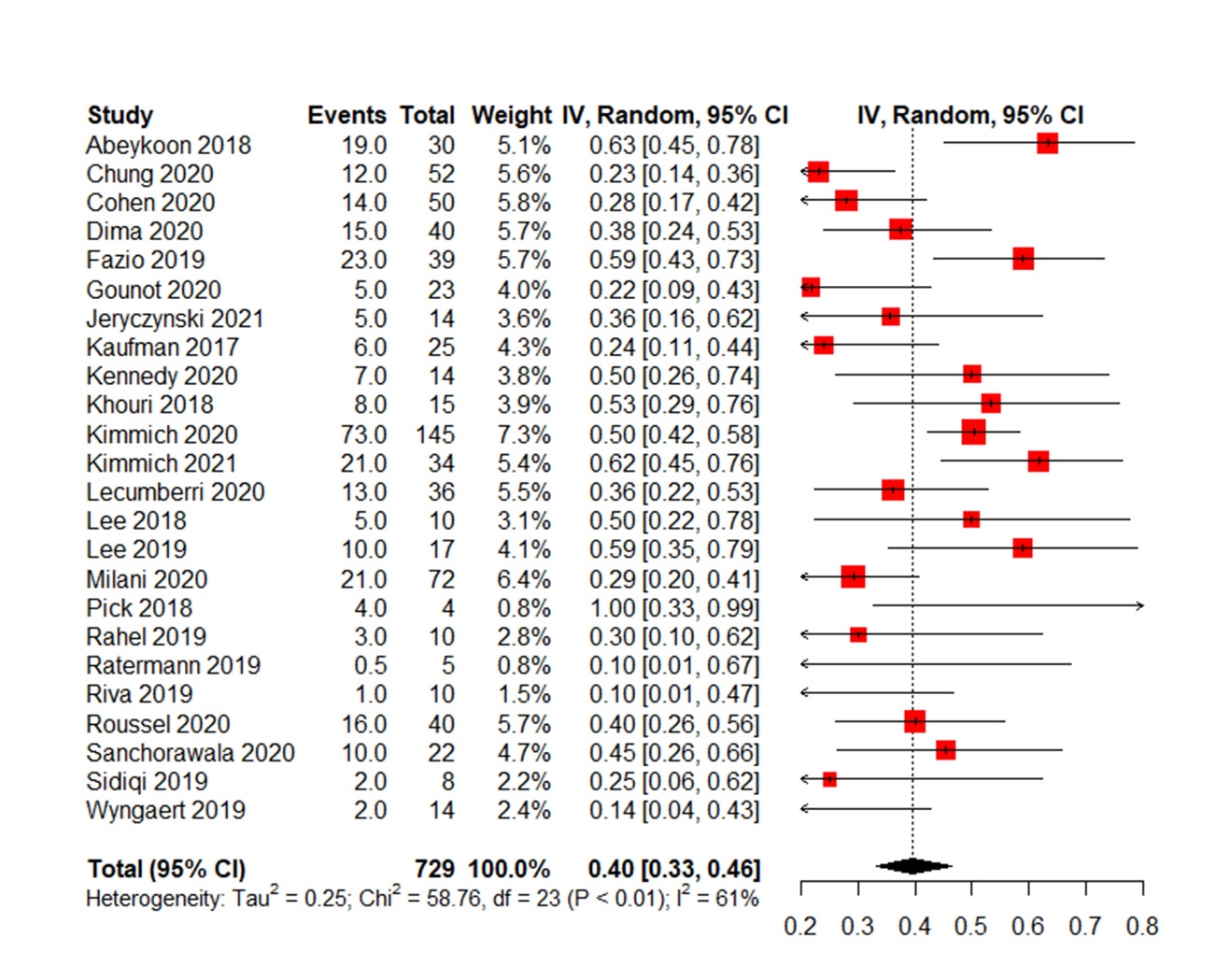
**Figure S3. Meta-analysis forest plot of very good partial response**


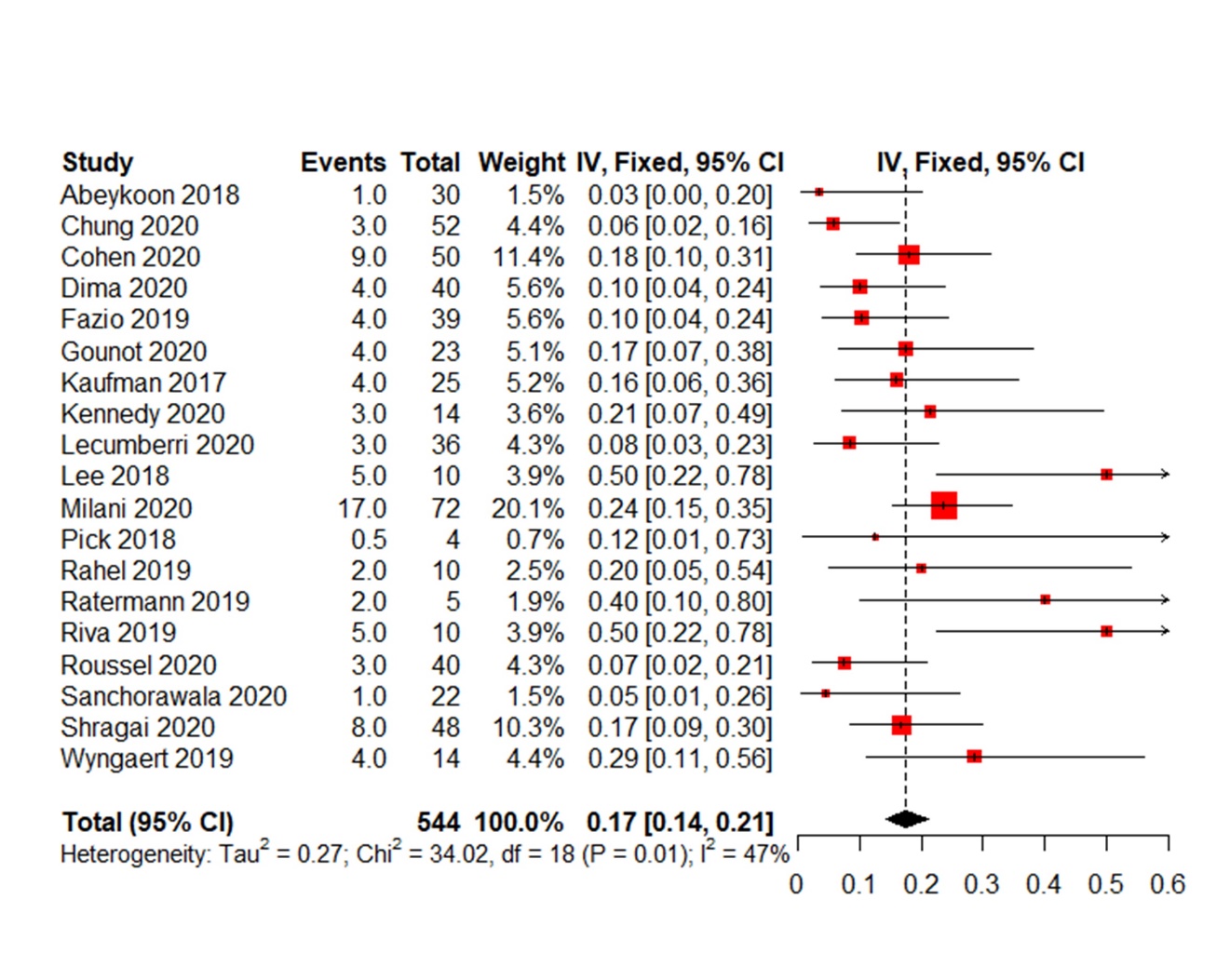
**Figure S4. Meta-analysis forest plot of partial response**


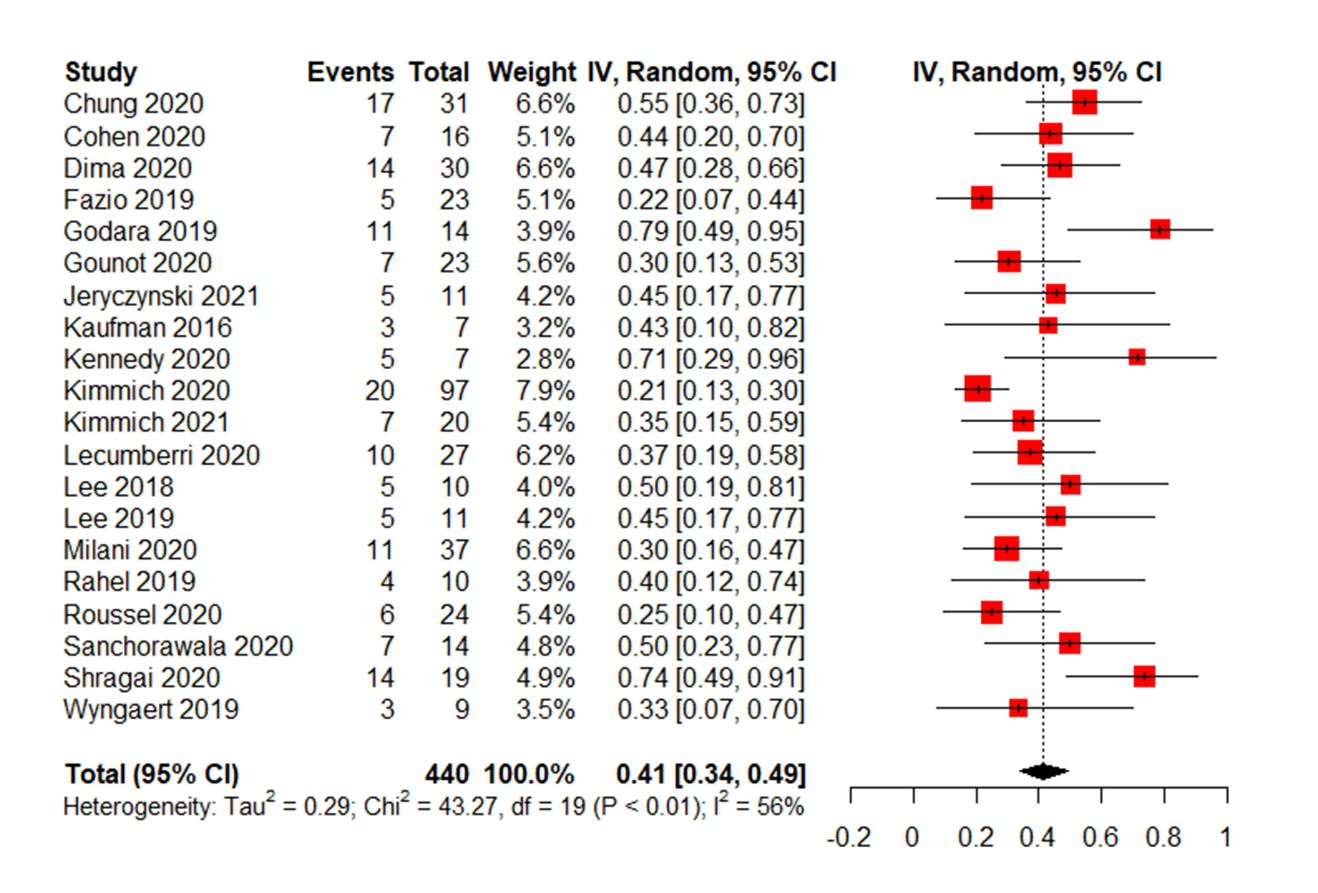
**Figure S5. Meta-analysis forest plot of cardiac response**


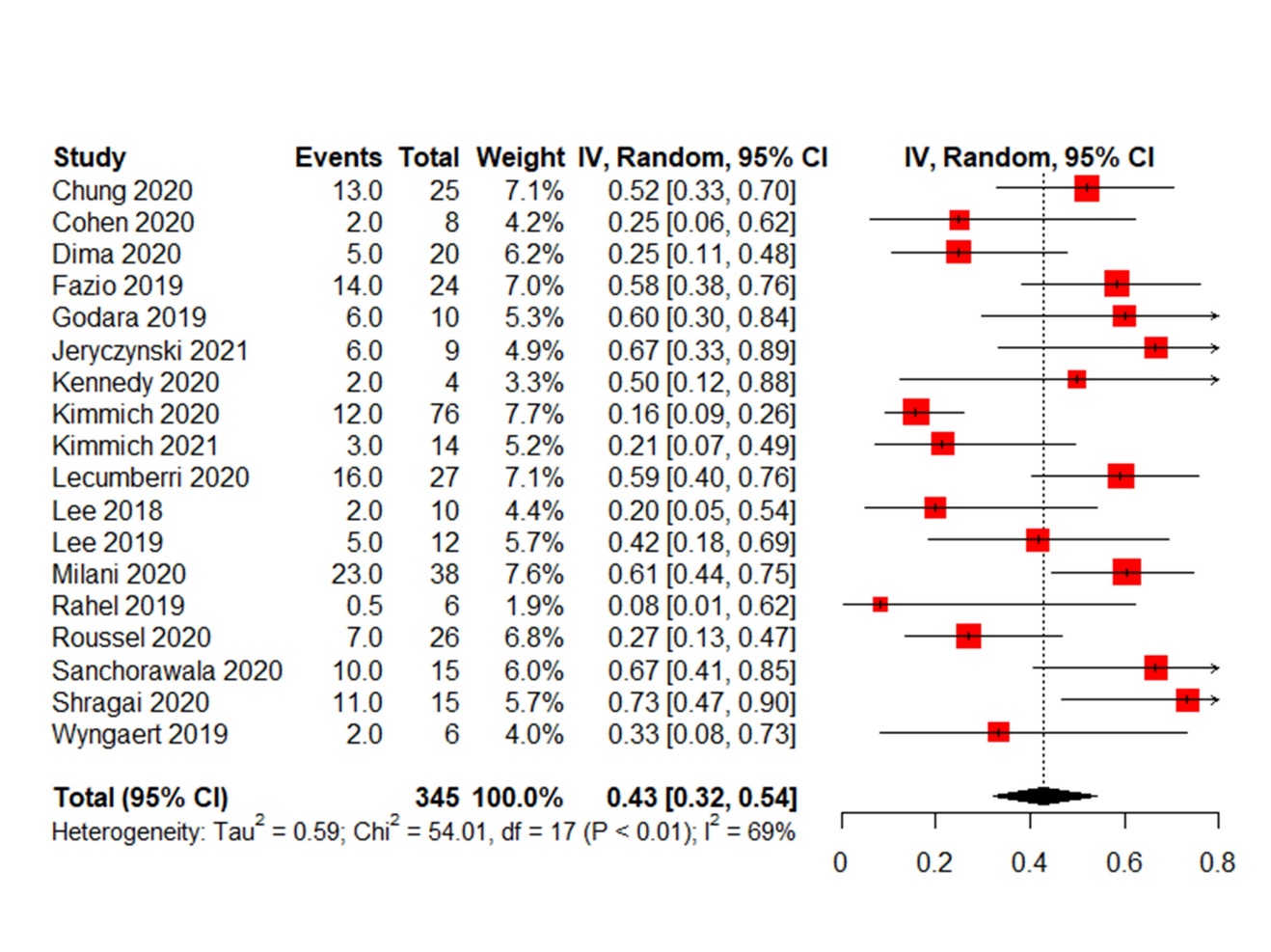
**Figure S6. Meta-analysis forest plot of renal response**


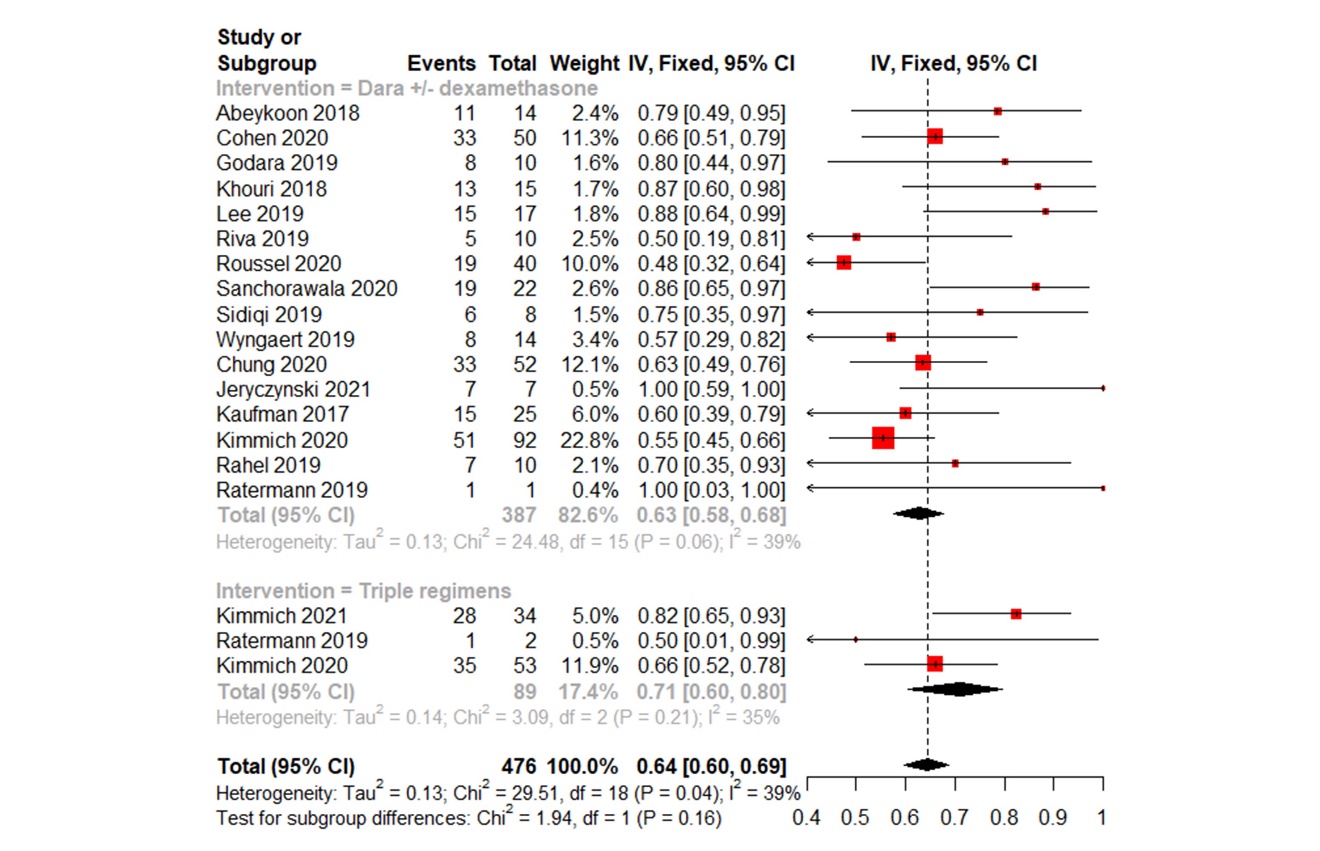
**Figure S7. Meta-analysis forest plot of ≥ VGPR-intervention**


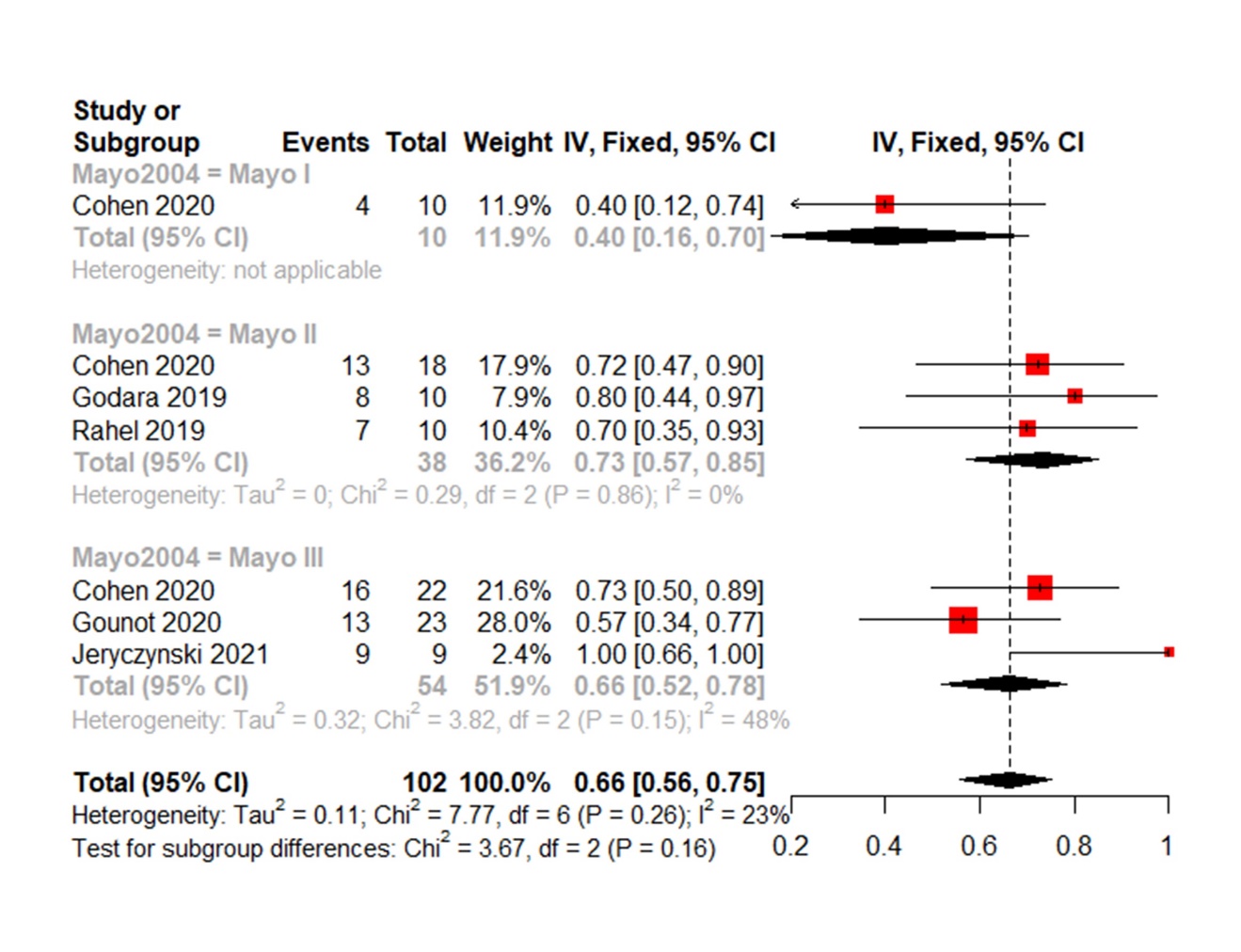
**Figure S8. Meta-analysis forest plot of ≥ VGPR-Mayo 2004**


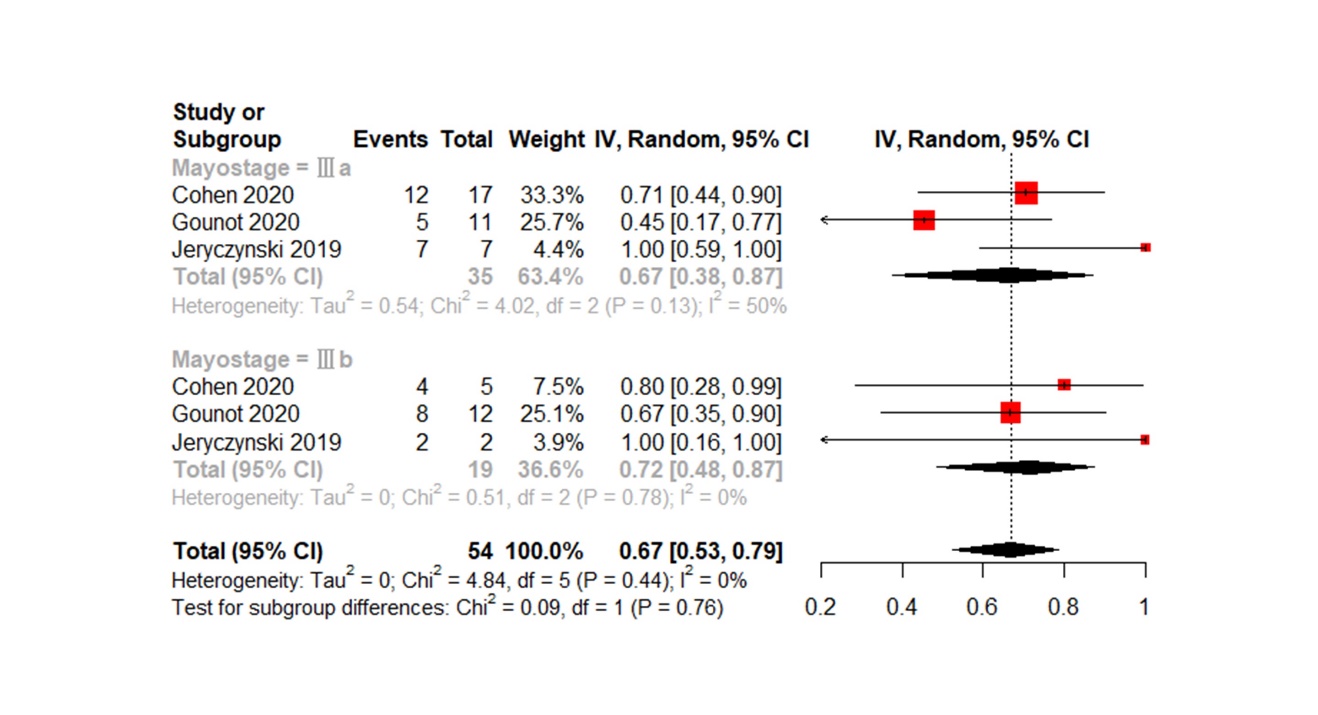
**Figure S9. Meta-analysis forest plot of ≥ VGPR-Mayo** **2004 ⅢA/B**



**Figure S10. Meta-analysis forest plot of ≥ VGPR-primary or secondary**


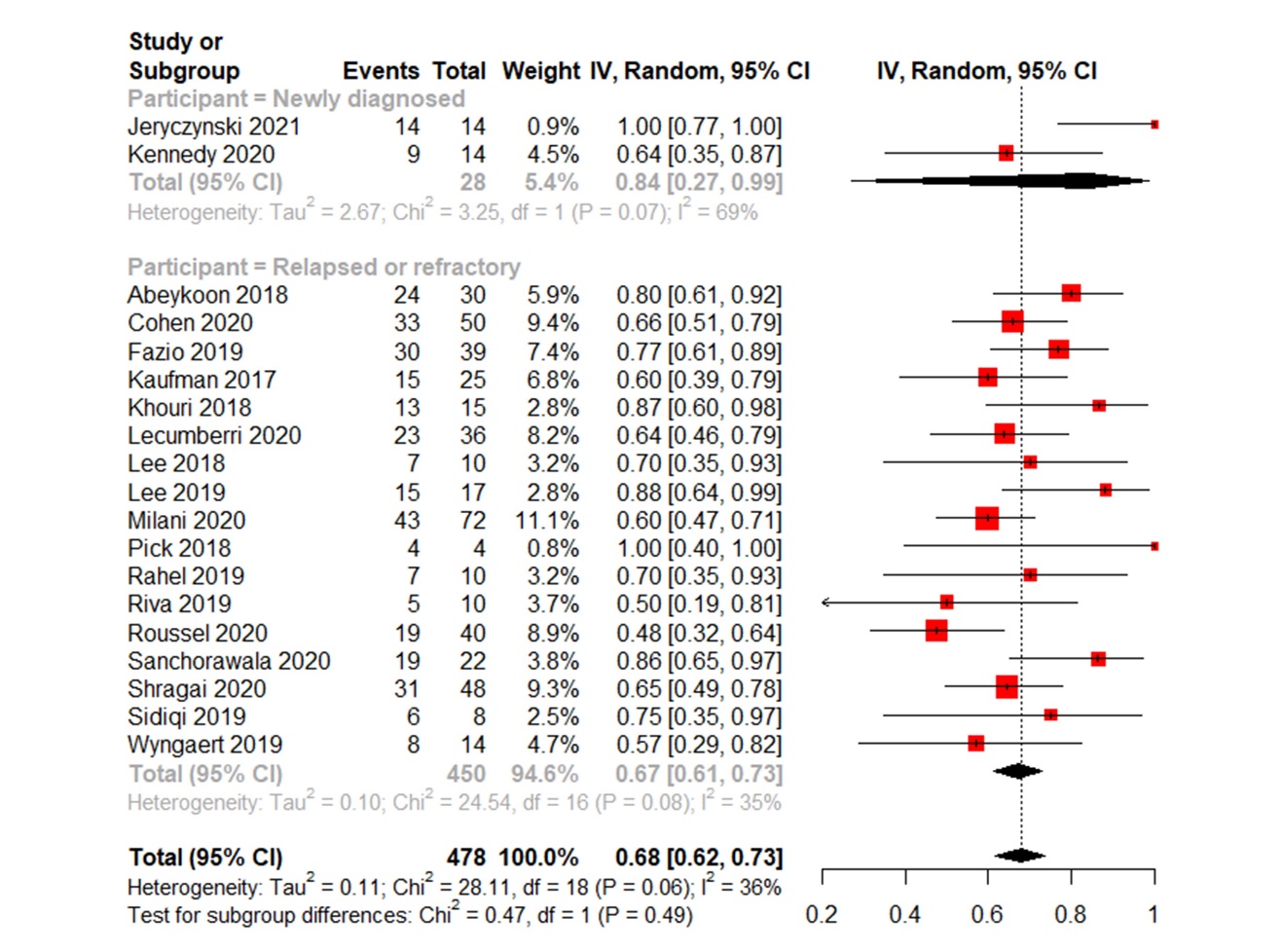
**Figure S11. Meta-analysis forest plot of ≥ VGPR-line of therapy**


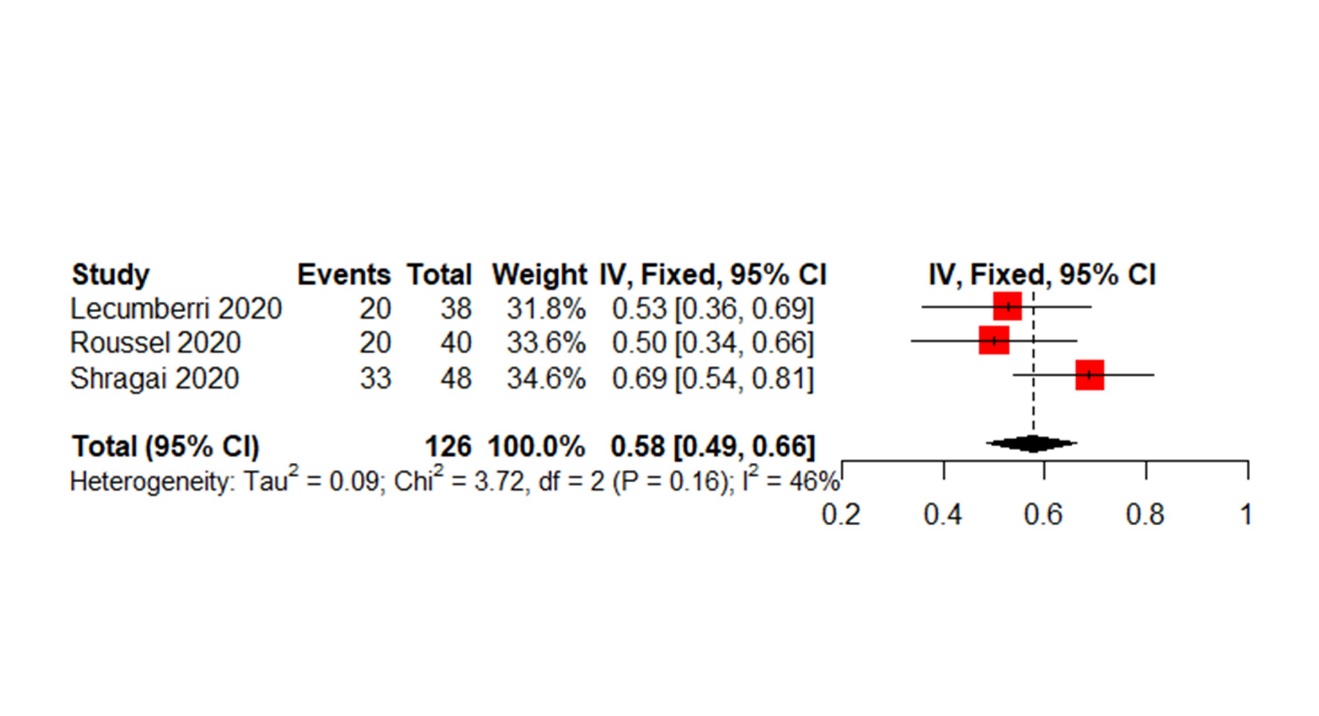
**Figure S12. Meta-analysis forest plot of PFS-1 year or longer**


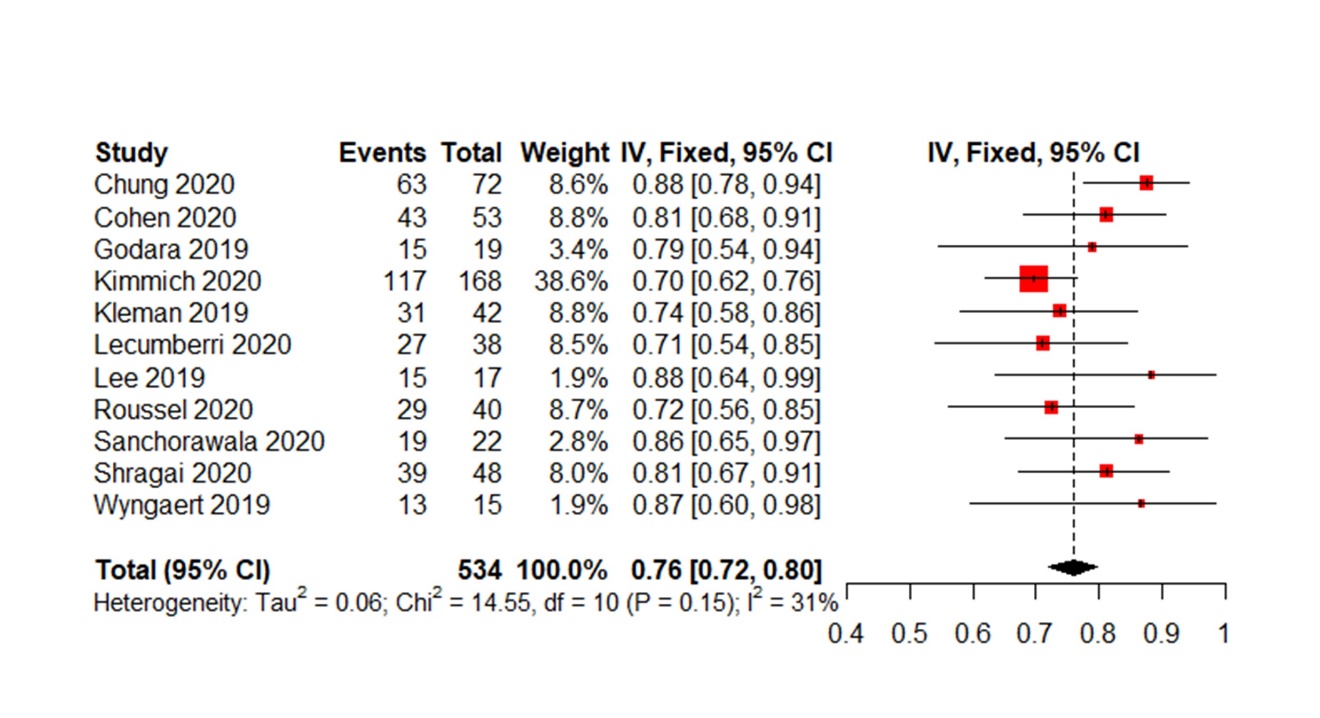
**Figure S13. Meta-analysis forest plot of OS-1 year or longer**


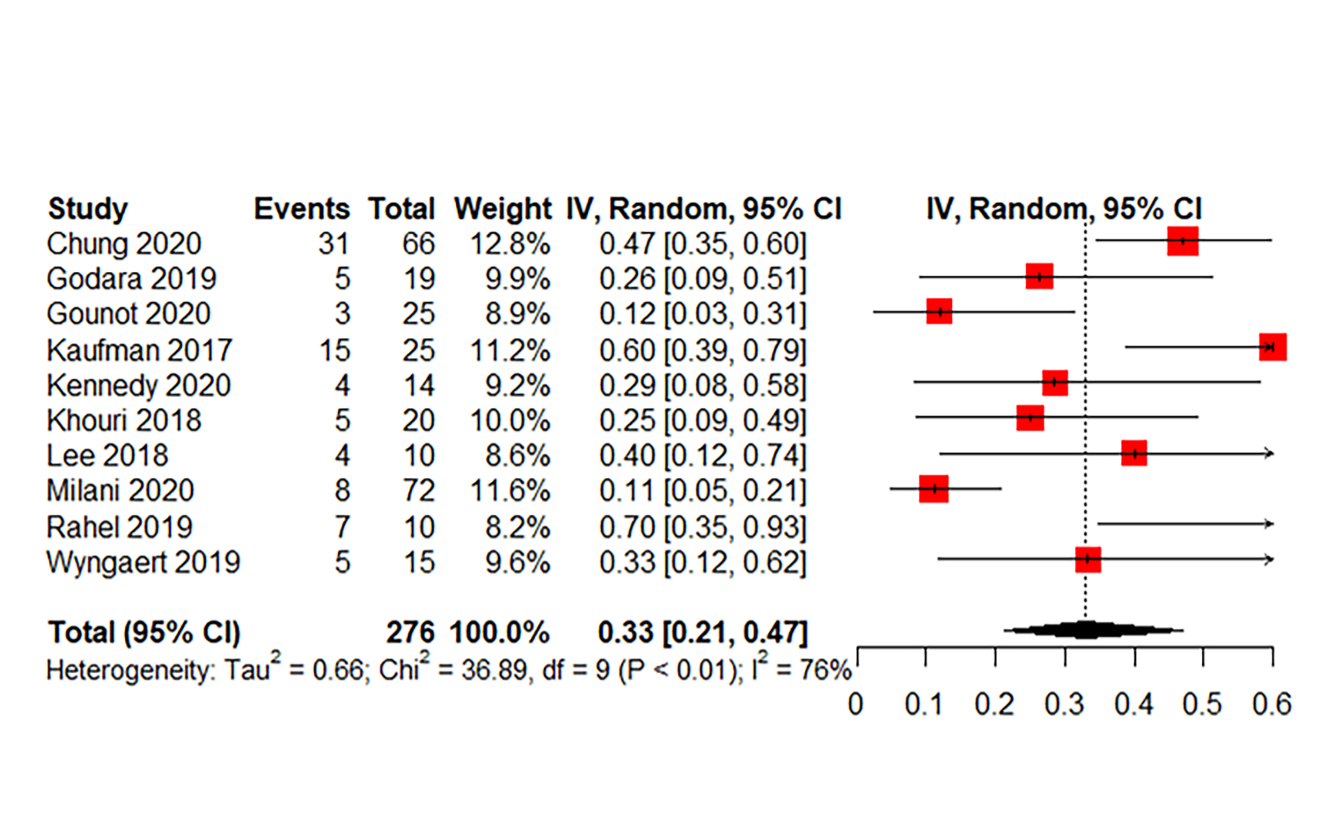
**Figure S14. Meta-analysis forest plot of Infusion related reaction-grade-1-2**


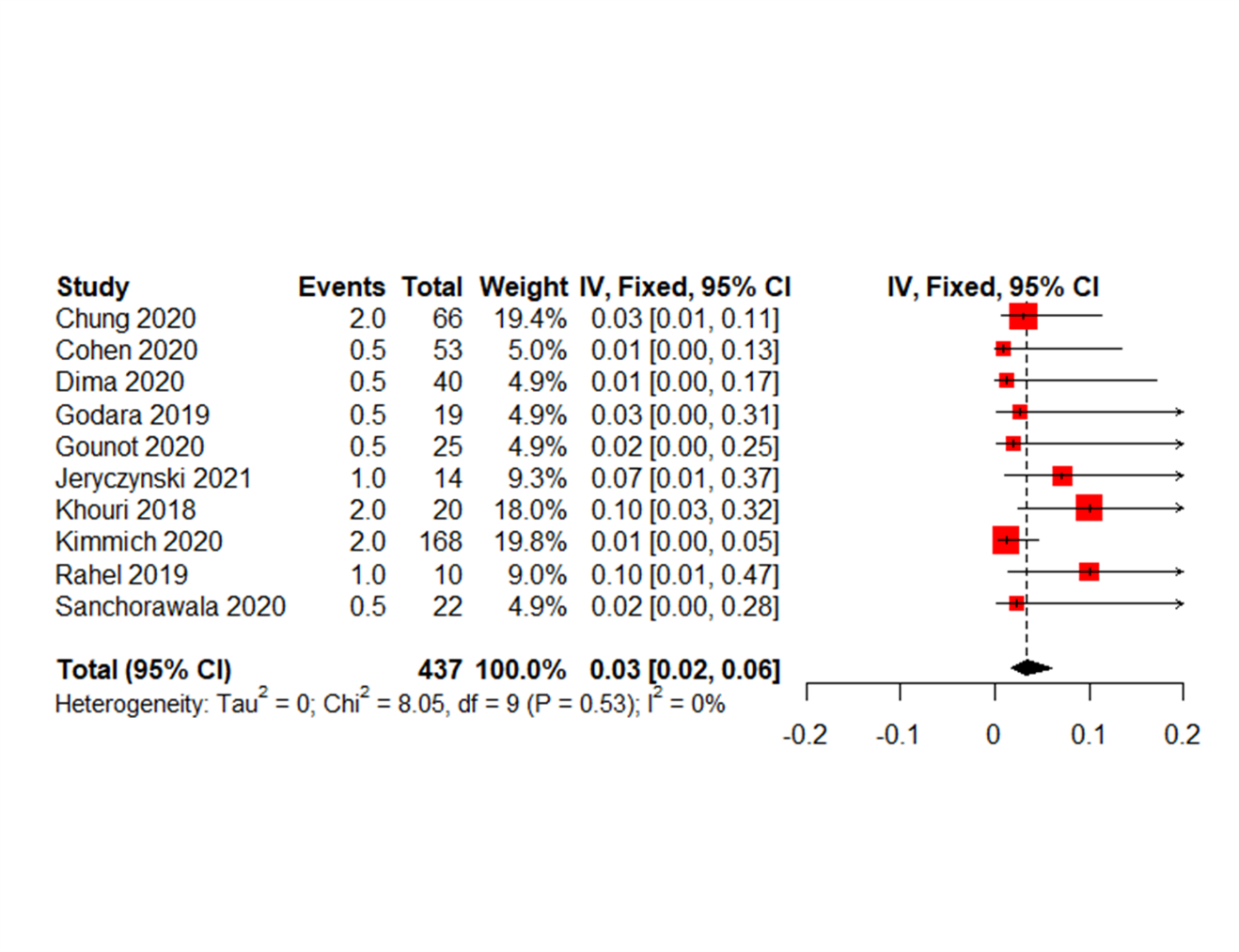
**Figure S15. Meta-analysis forest plot of Infusion related reaction-grade-3-4**


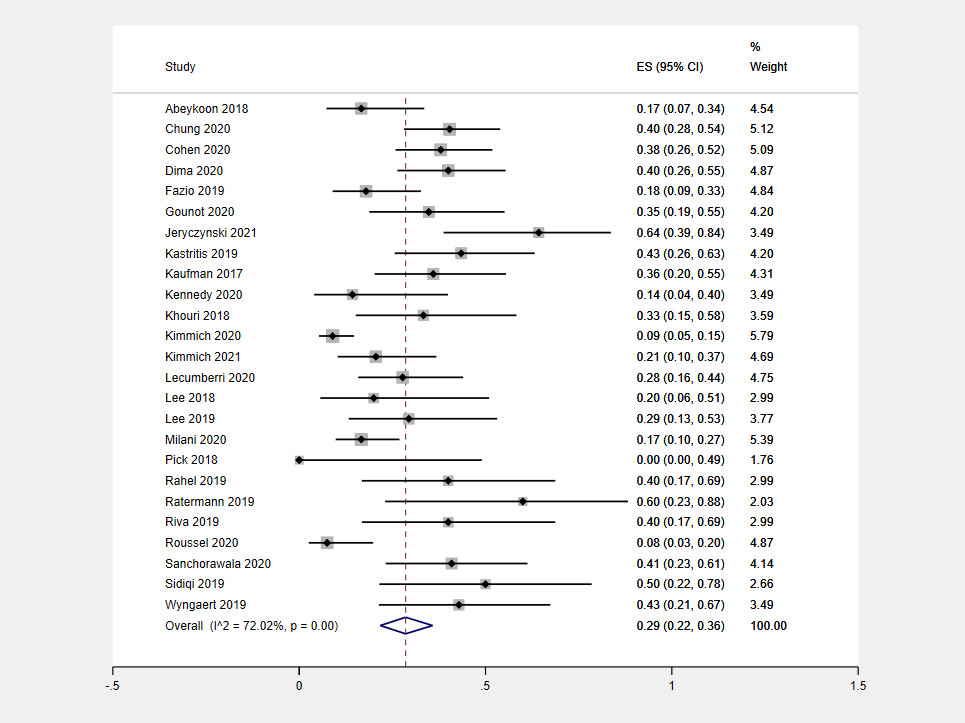
**Figure S16. Meta-analysis forest plot of complete remission**


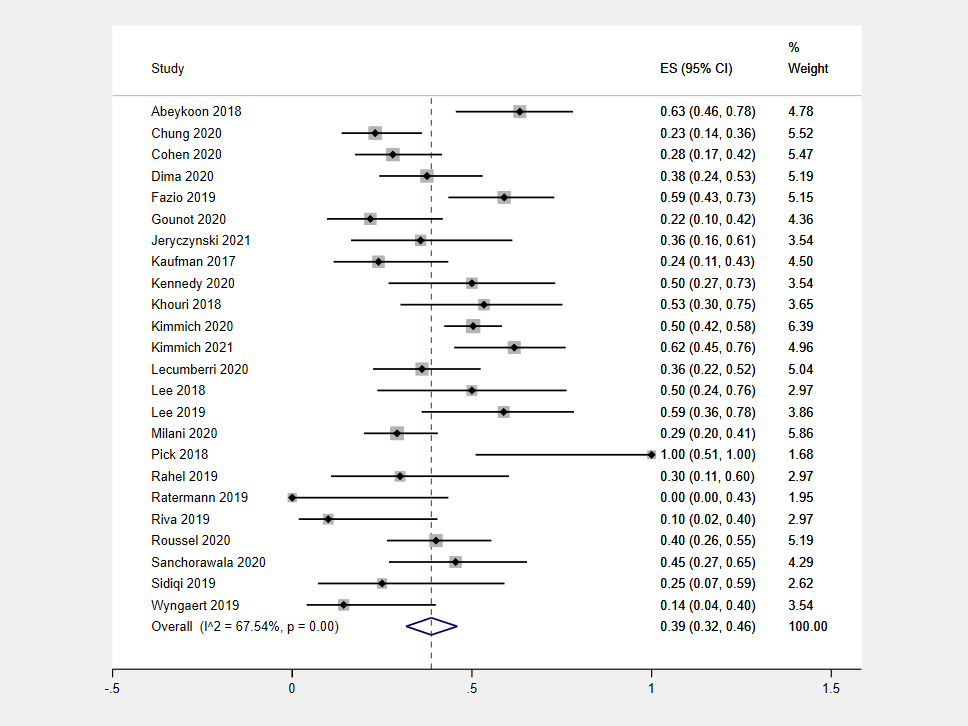
**Figure S17. Meta-analysis forest plot of very good partial response**


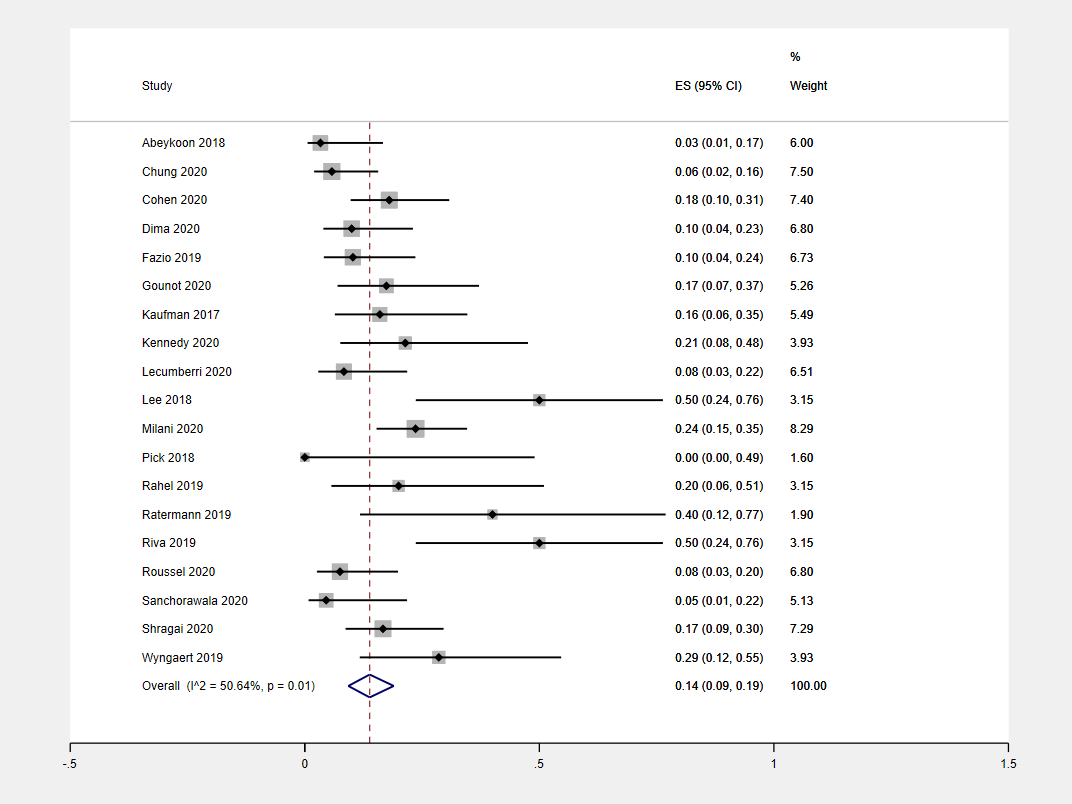
**Figure S18. Meta-analysis forest plot of partial response**


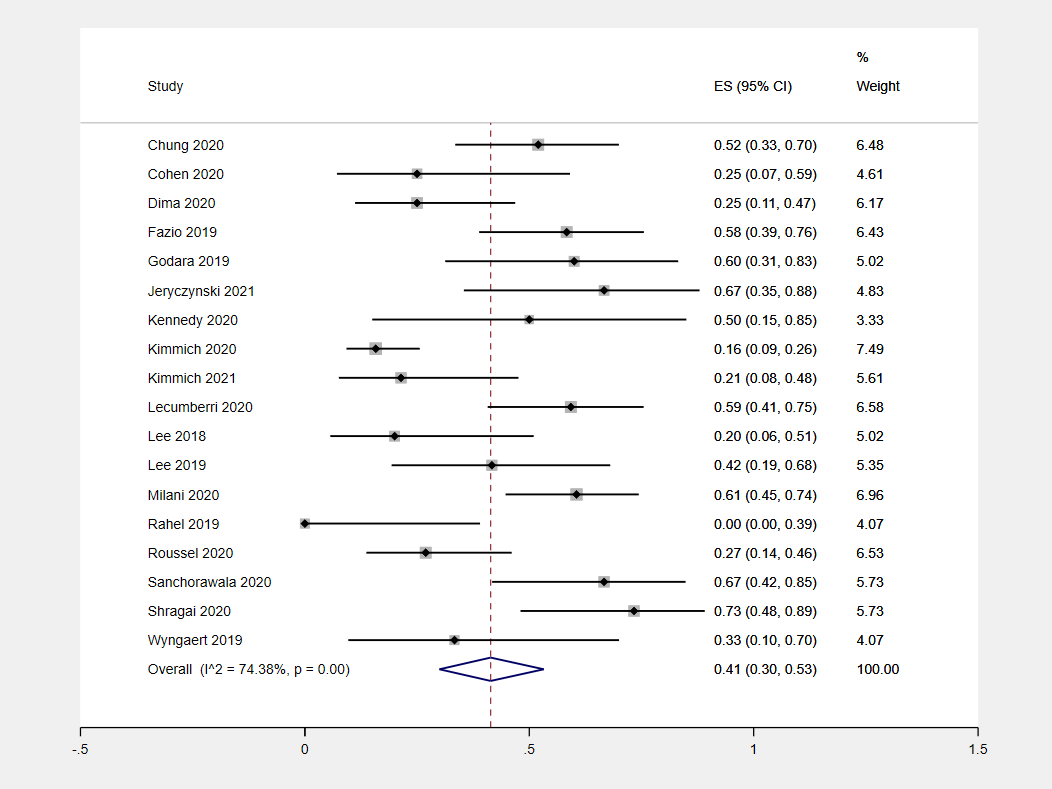
**Figure S19. Meta-analysis forest plot of Renal response**


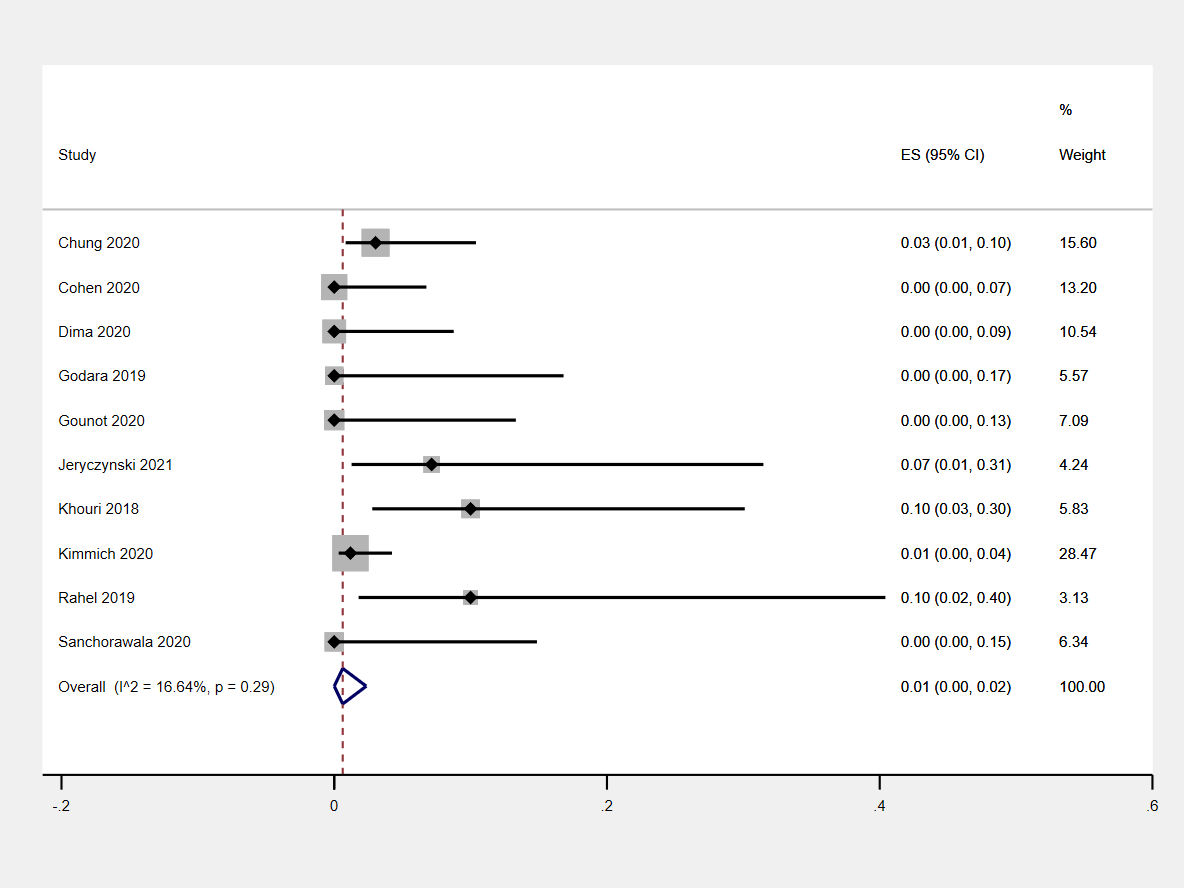
**Figure S20. Meta-analysis forest plot of Infusion related reaction-grade-3-4**
